# Climate mediates continental scale patterns of stream microbial functional diversity

**DOI:** 10.1186/s40168-020-00873-2

**Published:** 2020-06-13

**Authors:** Félix Picazo, Annika Vilmi, Juha Aalto, Janne Soininen, Emilio O. Casamayor, Yongqin Liu, Qinglong Wu, Lijuan Ren, Jizhong Zhou, Ji Shen, Jianjun Wang

**Affiliations:** 1 State Key Laboratory of Lake Science and Environment, Nanjing Institute of Geography and Limnology, Chinese Academy of Sciences, Nanjing, 210008 China; 2 Finnish Meteorological Institute, P.O. Box 503, FI-00101 Helsinki, Finland; 3 Department of Geosciences and Geography, University of Helsinki, 00014 Helsinki, Finland; 4 Integrative Freshwater Ecology Group, Centre of Advanced Studies of Blanes-Spanish Council for Research CEAB-CSIC, E-17300 Blanes, Spain; 5 University of Chinese Academy of Sciences, Beijing, 1000049 China; 6 Key Laboratory of Tibetan Environment Changes and Land Surface Processes, Institute of Tibetan Plateau Research, Chinese Academy of Sciences, Beijing, 100101 China; 7 Department of Ecology, Jinan University, Guangzhou, 510632 China; 8 Department of Microbiology and Plant Biology, Institute for Environmental Genomics, University of Oklahoma, Norman, OK 73019 USA; 9 State Key Joint Laboratory of Environment Simulation and Pollution Control, School of Environment, Tsinghua University, Beijing, 100084 China; 10 Earth Science Division, Lawrence Berkeley National Laboratory, California, 94270 USA

**Keywords:** Stream biofilm, Elevational gradients, Microbial functional genes, Macroecology, Climate change

## Abstract

**Background:**

Understanding the large-scale patterns of microbial functional diversity is essential for anticipating climate change impacts on ecosystems worldwide. However, studies of functional biogeography remain scarce for microorganisms, especially in freshwater ecosystems. Here we study 15,289 functional genes of stream biofilm microbes along three elevational gradients in Norway, Spain and China.

**Results:**

We find that alpha diversity declines towards high elevations and assemblage composition shows increasing turnover with greater elevational distances. These elevational patterns are highly consistent across mountains, kingdoms and functional categories and exhibit the strongest trends in China due to its largest environmental gradients. Across mountains, functional gene assemblages differ in alpha diversity and composition between the mountains in Europe and Asia. Climate, such as mean temperature of the warmest quarter or mean precipitation of the coldest quarter, is the best predictor of alpha diversity and assemblage composition at both mountain and continental scales, with local non-climatic predictors gaining more importance at mountain scale. Under future climate, we project substantial variations in alpha diversity and assemblage composition across the Eurasian river network, primarily occurring in northern and central regions, respectively.

**Conclusions:**

We conclude that climate controls microbial functional gene diversity in streams at large spatial scales; therefore, the underlying ecosystem processes are highly sensitive to climate variations, especially at high latitudes. This biogeographical framework for microbial functional diversity serves as a baseline to anticipate ecosystem responses and biogeochemical feedback to ongoing climate change.

Video Abstract

## Highlights

1. Functional gene alpha diversity monotonically declines towards high elevations and assemblage composition shows increasing turnover with greater elevational distances, these patterns being consistent across mountains, kingdoms and functional gene families.
2. Climate primarily explains the alpha diversity and compositional changes at mountain and continental scales, with local non-climatic predictors gaining more importance at mountain scale.
3. Under future climate scenarios, alpha diversity and assemblage composition show substantial changes across Eurasia, especially at mid- and high-latitudes.

## Background

Across terrestrial and aquatic habitats, microorganisms mediate many ecosystem processes [1, 2] crucially linked to climate through complex interactions and feedbacks [3]. Gaining insight into the large-scale patterns of microbial diversity is essential for predicting climate change impacts on ecosystems worldwide. The assessment of biodiversity responses to climate change by functional approaches can improve the quantitative and predictive power of ecological research [4]. However, functional biogeography remains largely unexplored for microbes [5], especially in freshwater ecosystems. The increasing availability of low-cost molecular methods, such as high-throughput sequencing and gene arrays [6, 7], allows linking microbial assemblages with ecosystem processes and enables the application of novel functional perspectives to classic microbial biogeography questions. Among these questions, elucidating the drivers that underlie the biogeographical patterns constitutes a key task.

Climate is among the main drivers shaping large-scale gradients in microbial diversity. For instance, temperature has been shown to drive the latitudinal gradient in diversity of planktonic marine bacteria [8] or the continental scale gradients in diversity and assemblage composition of functional genes involved in denitrification and nitrogen fixation across forest soils [9, 10]. Moreover, microbial functional gene diversity and composition can respond rapidly under warming and cooling scenarios, as demonstrated by experiments on soils [11, 12]. These findings are framed in the metabolic theory of ecology [13], and thus higher mutation and speciation rates are expected towards warmer areas in accordance with the kinetics of biological processes. Furthermore, precipitation can also drive large-scale gradients in microbial diversity by constraining above ground vegetation and soil moisture across bioclimatic zones [14, 15]. Nevertheless, the role of climate in shaping microbial functional diversity patterns remains scarcely addressed at large scales for a wide range of functional genes. This is especially true for freshwaters, the most threatened ecosystems on Earth [16], where studies over small spatial scales have demonstrated temperature to drive microbial diversity and composition in lakes [17] and streams [18], as well as to be positively related to functional diversity of biofilm bacteria in streams [19]. Meanwhile, the relationship between precipitation and freshwater microbial assemblages has been understudied although, for instance, rainfall seasonality is linked to functional diversity of stream biofilms [20]. At large geographical scales, precipitation may impact lake microbial assemblages as climate change-related phenomena are altering the nitrogen and phosphorus contents of water rainfall [21].

Here, we provide evidence from three mountainsides across Eurasia (Additional file 1: Figure S1) for climate to mediate large-scale gradients in stream microbial functional gene diversity. Firstly, we reveal the biogeographical patterns of functional diversity within and across mountainsides. We secondly determine the role of climatic and local non-climatic predictors, i.e. in situ abiotic conditions, in shaping such patterns. Finally, based on the anticipated link between functional diversity and climate [9, 10, 22, 23], we perform predictive models [24] to project the changes in functional diversity under future climate scenarios [25] across the Eurasian river network. Towards these aims, we focus on microbial functional genes from kingdoms archaea, bacteria and fungi which are involved in the cycling of carbon (C), nitrogen (N), phosphorus (P) and sulphur (S) as well as in stress-related processes (St). The metabolic potential of these functional genes was assessed by the DNA-based gene array GeoChip 4.0 [7], which contains approximately 82,000 probes and covers 141,995 gene sequences from 410 functional gene families. In total, we detected 15,289 functional genes linked to 88 functional gene families (Additional file 1: Table S1) included in these five functional categories (C, N, P, S and St). Microbial functional genes are ideal candidates for biodiversity projections under future climates as they meet two essential requirements of spatial distribution modelling [26], such as a high equilibrium with the environment because of their rapid response to changing conditions [11, 12], and the relatively high dispersal capacity reported for microbes [27]. Moreover, literature indicates the utility in using elevational gradients for anticipating biodiversity responses to climate change over large geographical extents [28]. Our study constitutes a remarkable contribution to the emerging field of functional biogeography [5], as most previous studies examined the large-scale patterns of microbial diversity from taxonomic or phylogenetic perspectives [8, 29].

## Results and discussion

**Fig. 1:**
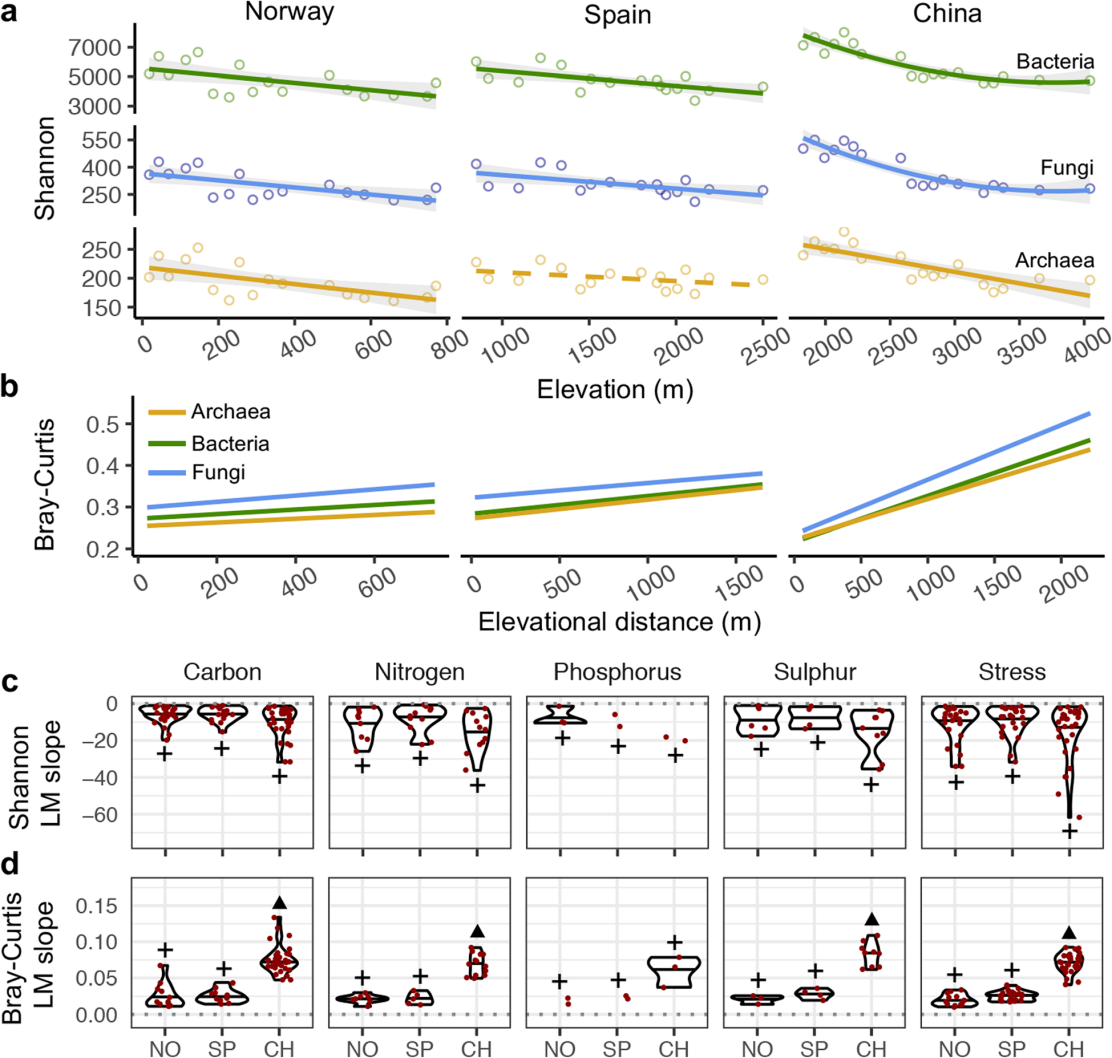
Responses of functional gene alpha diversity and compositional turnover to elevation. The relationships between Shannon diversity and elevation (**a**, **c**) were examined by linear models, and the model significances were determined with F-statistics (*P* < 0.05). For kingdoms (**a**), we considered linear and quadratic terms and selected the best models, i.e. those that minimized the corrected Akaike’s information criterion. The adjusted *R*^2^ values were 0.394, 0.181 and 0.672 for archaea, 0.346, 0.394 and 0.829 for bacteria, and 0.428, 0.346 and 0.844 for fungi, in Norway, Spain and China, respectively. The relationships between Bray-Curtis dissimilarity and elevational Euclidean distance (**b**, **d**) were calculated by linear models, and the model significances were obtained by a Mantel test (1000 permutations, *P* < 0.05). For kingdoms (**b**), the Pearson *r* values were 0.212, 0.361 and 0.643 for archaea, 0.199, 0.28 and 0.700 for bacteria and 0.283, 0.25 and 0.692 for fungi, in Norway, Spain and China, respectively. Across functional categories, we show the slope values from LMs assessing the gene family alpha diversity-elevation relationships (**c**) and the gene family compositional turnover-elevational distance relationships (**d**). For kingdoms (**a**, **b**), significant and non-significant models are shown as solid and dashed lines, respectively. The violin boxplots for the Shannon diversity (**c**) and Bray-Curtis dissimilarity (**d**) depict the median and the first and the third quartiles of the slopes of the gene families with significant (*P* < 0.05) models. Across functional categories, differences among mountains in the model slopes for the Shannon diversity (**c**) and Bray-Curtis dissimilarity (**d**) were examined with a Bonferroni-corrected pairwise *t*-test (*P* < 0.05) and are indicated with the symbols + and ▲. The elevation (**a**) and elevational distances (**b**) are shown as raw data for visualization purposes (*z*-transformed for the analyses). C, carbon; N, nitrogen; P, phosphorus; S, sulphur; NO, Norway; SP, Spain; CH, China. Details on the models can be found in methods

Within mountains, functional gene alpha diversity presented a significant (linear model, LM; *P* < 0.05) monotonic decline towards high elevations (Fig. 1a, Additional file 1: Table S2) and assemblage composition showed a significant (*P* < 0.05) increase in turnover with greater elevational distances (Fig. 1b, Additional file 1: Table S2). These patterns were highly consistent across mountains and kingdoms and, interestingly, concur with recent reports showing that tree functional diversity declines with elevation in forests worldwide [22]. The uniformity in our alpha diversity gradients contrasts with the variety of elevational trends reported for microorganisms on taxonomic or phylogenetic approaches in both soil [30] and freshwater [31] environments. For instance, species richness of stream bacteria showed hump-shaped, monotonically increasing and U-shaped patterns on the same mountainsides in Norway, Spain and China, respectively [31]. Such disparities between microbial taxonomic and functional diversities are an interesting phenomenon likely caused by functional redundancy which seems an inevitable outcome in open microbial systems where diversity is not limited by low immigration rates [32]. Meanwhile, the consistency in our compositional turnover gradients agrees with the general spatial distance-decay patterns documented for microbial taxonomic composition [33] and with the elevational distance-decay patterns observed for microbial functional gene assemblages in soils [34]. When scaling down to functional gene families, most of them also showed a significant (LM; *P* < 0.05) decline in alpha diversity with elevation (Fig. 1c, Additional file 1: Figure S2a), and this pattern was congruent across the three mountains for 58% of all families (51 out of 88). Taken together, these findings highlight a general response of stream microbial functional diversity to elevation across mountains, kingdoms and functional gene families.

Despite the remarkable congruency in our elevational patterns, the mountain in China showed the strongest trends for both functional gene alpha diversity and assemblage composition. Such a finding was consistent across kingdoms and functional gene families. For instance, regarding gene families, China showed the highest proportion of significant (*P* < 0.05) and the strongest (Bonferroni-pairwise *t*-test; *P* < 0.05) elevational gradients in alpha diversity and composition (Fig. 1c, d and Additional file 1: Figures S2a and S2b). These cross-mountain variations in the elevational trends might be related to distinct functional gene regional pools and latitudes [35] as the tropospheric temperature lapse rate decreases from the equator to the poles [36]. Beyond the differences in slope steepness of our diversity gradients, the high congruency in the elevational trends both across kingdoms and gene families constitutes an interesting finding. For instance, the cross-family consistency in the elevational trends suggests that the microbial-mediated stream processes related to biogeochemical cycling and stress are constrained by similar driving forces.

Across mountains, functional gene assemblages exhibited continental scale patterns and regional differences on the overall gene pool were detected in alpha diversity (analysis of variance, ANOVA; *P* = 0.001; Additional file 1: Table S3) and assemblage composition (permutational analysis of variance; *R*^2^ = 0.387, *P* = 0.001; Table S3). The streams in China showed the highest mean alpha diversity (Bonferroni-corrected pairwise *t*-test; *P* < 0.05; Additional file 1: Table S3) as well as the most different composition (Additional file 1: Table S3 and Fig. S3a) for the overall functional gene pool, such a result being consistent across the three kingdoms. These findings expand the large-scale gradients in functional diversity documented for both macro- [22] and microorganisms [6, 9, 10] from the terrestrial to the freshwater realm and, namely for microbial functional gene diversity, from genes linked to denitrification [9] and nitrogen fixation [10] to genes involved in other biogeochemical cycles (e.g. C, P and S) and St-related processes.

**Fig. 2:**
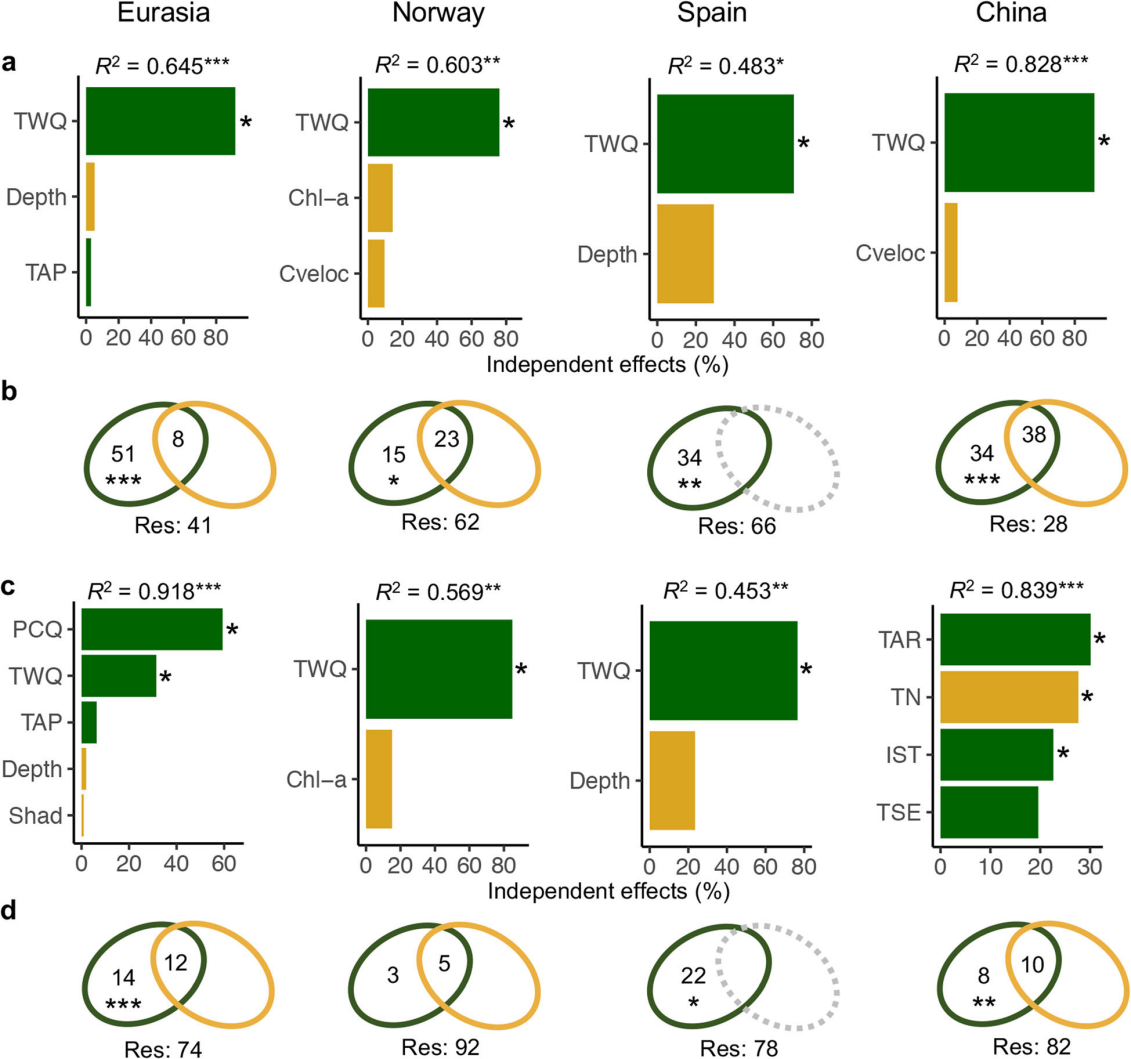
Relative contributions of climatic and local non-climatic predictors in shaping the functional gene alpha diversity and assemblage composition. The independent effects of the selected predictors on the Shannon diversity (**a**) and assemblage composition (**c**) were examined by hierarchical partitioning, and their significance (*P* < 0.05) was tested through a 1000 randomization-based procedure. The *R*^2^ and *P* values above the plots in (**a**) and (**c**) were calculated by linear models. The variances in Shannon diversity (**b**) and assemblage composition (**d**) associated with the climatic and local non-climatic predictors were obtained using variation partitioning, based on adjusted *R*^*2*^ and significances tested with analysis of variance. As local predictors were not selected in Spain, the variance associated with climatic predictors was determined through linear models for the Shannon diversity and a Mantel test (1000 permutations) for the assemblage composition, with test significance based on F-statistic and Pearson *r* value, respectively. The significance levels in the variation partitioning are indicated by *P < 0.05, **P < 0.01, ***P < 0.001. The assemblage composition was estimated using first axis coordinates from principal coordinate analysis (PCoA) based on Bray-Curtis dissimilarity matrices (see Fig. S3 in Additional file 1 for details on the axis-explained variance). Green and yellow bars represent climatic and non-climatic local predictors, respectively. Details on predictor selection are presented in “Methods” and in Additional file 1 (Fig. S14). TWQ, mean temperature of the warmest quarter; TAP, total annual precipitation; PCQ, mean precipitation of the coldest quarter; TAR, temperature annual range; IST, isothermality (relationship between air temperature diurnal and annual ranges); TSE, temperature seasonality; Chl-a, chorophyll-*a*; Cveloc, current velocity; Shad, shading; TN, total nitrogen

To elucidate the drivers shaping our functional diversity gradients, we examined a set of 19 climatic predictors comprising the average conditions for years 1960–1990 and 11 local non-climatic predictors reported as important drivers of microbial diversity [37, 38] (see “Methods” for details). Regarding alpha diversity, we found that mean temperature of the warmest quarter (TWQ) was the most important predictor at both continental and mountain scales, revealed by hierarchical partitioning [39] (HP; Fig. 2a), LMs (Additional file 1: Table S4) and random forest [40] (RF; Additional file 1: Figure S4a). Such a finding is in line with the biological importance of the growing season conditions in mountains [41]. Importantly, the alpha diversity response to the TWQ at continental scale was consistent across mountains; that is, such a response was not affected by site location (TWQ, *P* < 0.001; latitude, *P* = 0.109; TWQ × latitude, *P* = 0.573). Such a positive relationship between temperature and stream microbial diversity have been also reported using taxonomic approaches, e.g. in a recent study on streams from the Rocky Mountains [18]. In accordance with the metabolic theory of ecology [13], this large-scale temperature dependence of functional diversity could be associated with increasing mutation and speciation rates towards warmer areas [8–10]. This result may be also compatible with the energy hypothesis, which proposes that higher diversity coexists with higher available energy [42]. The consistent relationships between elevation and alpha diversity for most of the functional gene families further suggest a general temperature dependence for multiple stream processes. This finding was especially noticeable for families associated with stress processes (Additional file 1: Figure S5a) and highlights the need for future research on temperature dependence to consider a wider range of ecosystem processes in addition to mere C-cycling [43].

Regarding assemblage composition, temperature-related variables constituted the best predictors at mountain scale (Fig. 2c; Additional file 1: Table S4). This result is in line with previous studies on terrestrial habitats, which show changes in functional gene composition across temperature gradients [9, 10]. Across mountains, however, mean precipitation of the coldest quarter (PCQ) was consistently retained as the best continental scale predictor in HP (Fig. 2c), LMs (Additional file 1: Table S4), generalized dissimilarity models [44] (GDMs; Additional file 1: Figure S6a) and RF (Additional file 1: Figure S4b). The PCQ, i.e. winter precipitation, mediates the hydrology in high mountains through the accumulation of snow, consequently affecting local factors shaping the spatial patterns of stream microbial assemblages [31, 45, 46]. However, the high predictive power of the PCQ only at continental scale suggests a link between this factor and functional gene composition via hydrological mechanisms and agrees with the importance of precipitation when the spatial scale spans multiple biomes [47]. Such a finding was further supported by the effect of site locations on assemblage composition (LM; PCQ, *P* < 0.001; latitude, *P* < 0.001, PCQ × latitude, *P* = 0.183). These results agree with previous observational and experimental studies in soils, which show precipitation to be a large-scale driver of microbial assemblage composition by constraining vegetation types across biomes [14, 15].

Compared to climate, local non-climatic predictors explained a lower amount of geographical variation in alpha diversity and assemblage composition at both mountain and continental scales, with local non-climatic predictors gaining more importance at mountain scale as revealed by various statistical methods, such as variation partitioning (Fig. 2b, d). These results contrast with the importance of local non-climatic factors, such as nutrients or pH, in shaping freshwater microbial taxonomic diversity [48]. It suggests different driving forces for microbial taxonomic and functional gene diversities, which is in line with the decoupling observed between taxonomy and function in microbial systems [32]. For example, no local predictor was consistently meaningful for alpha diversity at neither continental nor mountain scales under the HP and LM analyses (Fig. 2a; Additional file 1: Table S4). Regarding composition, only total nitrogen was identified as a significant driver in China. Interestingly, nitrogen availability has been shown to elicit changes in microbial assemblage composition, but not in alpha diversity, under elevated nutrient inputs in a global experiment on grasslands [49]. Other potentially important predictors of freshwater microbial diversity, such as those informing about carbon availability [48], may also play a role. For instance, dissolved organic matter poorly predicts taxonomic elevational diversity and the biogeography of freshwater bacteria [31, 50], but is associated with bacterial trait structure over large spatial scales as a response to the terrestrial influence gradient in a continuum of small streams to large lakes [50]. We therefore encourage further studies to assess the role of carbon availability in explaining functional gene diversity of stream microbes.

Collinearity between climatic and local non-climatic predictors is frequent for elevational gradients. By including three different mountainsides, we could test the role of all local non-climatic predictors across different models, either based on individual mountains or the overall dataset, and found climatic predictors to primarily mediate both alpha diversity and assemblage composition (see RF results on all predictors included in the full models at Additional file 1: Figure S7). For instance, among all local non-climatic predictors, only conductivity was revealed as a candidate to importantly drive alpha diversity when the entire dataset was analysed (see RF results prior to the removal of highly correlated predictors at Additional file 1: Figure S8). However, when each mountain was analysed separately, alpha diversity showed a significant and cross-mountain consistent response only to TWQ (LM; *P* < 0.05 for all mountains; Additional file 1: Figure S9), but not to conductivity (LM; *P* = 0.732 for Norway; Additional file 1: Figure S9) or other relevant local non-climatic predictors (Additional file 1: Fig. S9). Furthermore, we assessed all possible models including either the best climatic predictors or conductivity and their respective interactions with latitude to control for cross-mountain consistency and found that best models based on Akaike weights [51] were those containing the climatic predictors and the interaction with latitude (weight = 0.725 for alpha diversity; weight = 1.000 for assemblage composition; Additional file 1: Table S5). Despite these evidences, we encourage further complementary manipulative experiments [28] to elucidate how functional gene diversity response to those local non-climatic predictors that covary or interact with climate, such as conductivity or nutrient loads [52]. Collectively, our findings show climate to mediate overall functional gene diversity in freshwater ecosystems as large spatial scale, suggesting that ongoing climate change can affect stream processes worldwide.

**Fig. 3:**
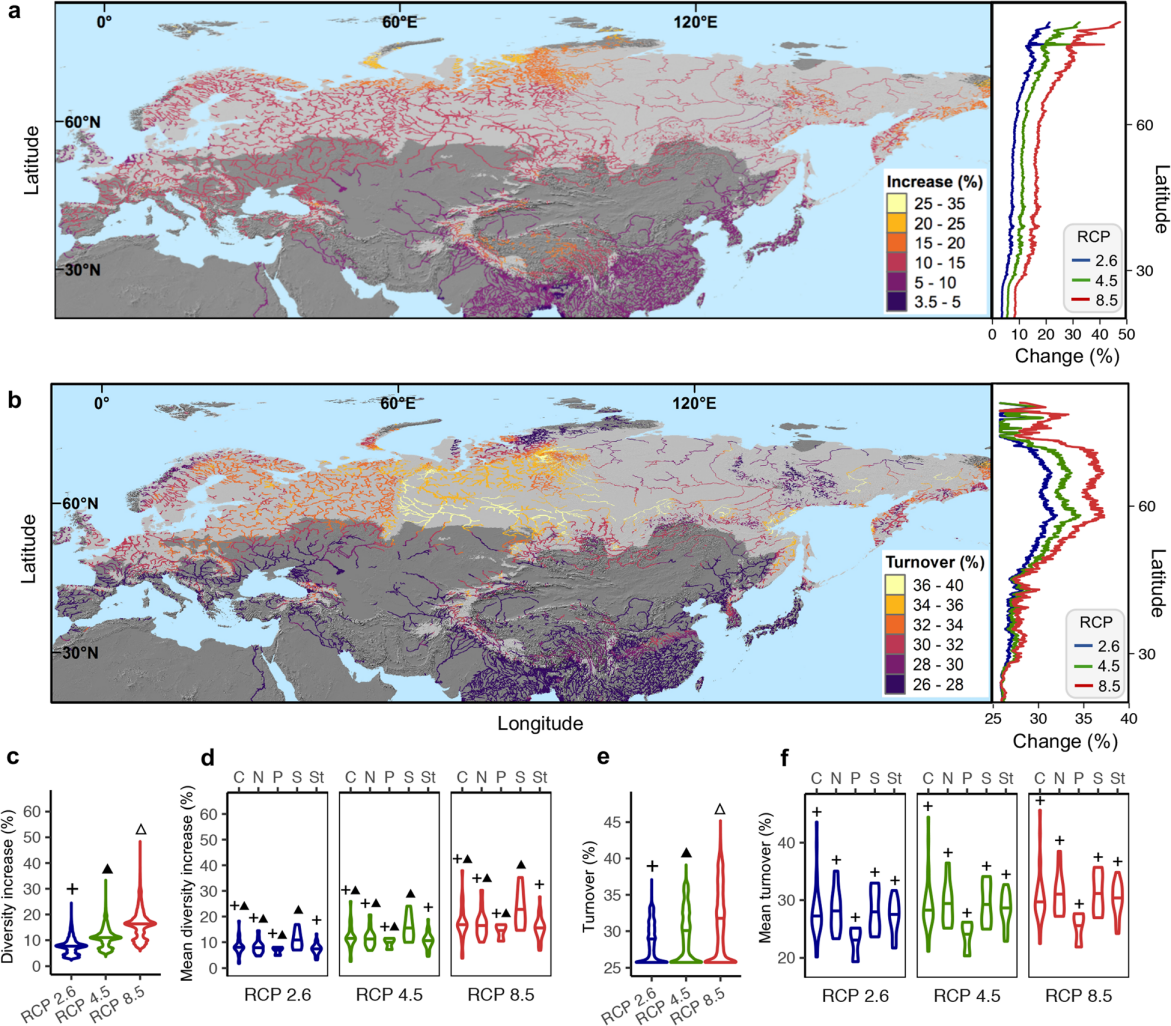
Projected changes across the Eurasian river network in functional gene alpha diversity and assemblage composition under future climate scenarios. The relative increase in Shannon diversity assuming the moderate emission scenario RCP 4.5 (**a**, map) and the relative increase in Shannon diversity averaged by latitude for the three emission scenarios (**a**, line plot) were calculated by linear models using temperature of the warmest quarter (TWQ) and precipitation of the coldest quarter (PCQ) as predictors (*R*^2^ = 0.543; *P* < 0.001). The turnover rates assuming the moderate emission scenario RCP 4.5 (**b**, map) and the turnover rates averaged by latitude for the three scenarios (**b**, line plot) were calculated using generalized dissimilarity models with the TWQ and PCQ as predictors on Bray-Curtis dissimilarity matrices (*D*^2^ = 65.6%; *P* < 0.001). The violin boxplots show the median and the first and the third quartiles for the relative increase in Shannon diversity (**c**) and the turnover rate (**e**) on the overall gene pool and for the mean relative change in Shannon diversity (**d**) and the mean turnover rate (**f**) on every gene family pool, grouped into functional categories. Pairwise differences across functional categories regarding the relative change in the Shannon diversity (**d**) and turnover rates (**f**) were examined by a Bonferroni-corrected pairwise *t*-test (*P* < 0.05) post hoc analyses and are indicated with the symbols +, ▲ and △. Light and dark grey areas in maps depict the climate envelope covered by and extrapolated from the in situ data, respectively, in terms of TWQ and PCQ. C, carbon; N, nitrogen; P, phosphorus; S: sulphur; St, stress

Given that elevational gradients offer excellent possibilities to conduct natural experiments for assessing climate change impacts [28], we employed LMs and GDMs to project the trends of alpha diversity and assemblage composition, respectively, under future climate scenarios (period 2061–2080) across the Eurasian river network. We selected three scenarios from the Fifth Assessment Report of the Intergovernmental Panel on Climate Change [25]. They are based on the representative concentration pathways (RCP) 2.6, 4.5 and 8.5 and symbolize the most “optimistic”, one intermediate and the most “pessimistic” expectations of greenhouse emission rates, respectively, by the end of this century [53]. For modelling, we combined together the TWQ and PCQ as predictors, i.e. the variables that best explained the continental scale gradients in overall alpha diversity and assemblage composition, respectively. These predictors jointly explained a large amount of variance for both overall alpha diversity (LMs; *R*^2^ = 0.543; *P* < 0.001) and composition (GDMs; *D*^2^ = 65.6%; *P* < 0.001; Additional file 1: Figure S6b). Our projections show a general increase in alpha diversity (Fig. 3a, Additional file 1: Figure S10) and elevated compositional turnover rates (Fig. 3b, Additional file 1: Figure S11) as a response to future climates. Compared to the baseline, the median increase in alpha diversity and the median turnover rates were predicted to be 11.0% and 29.6%, respectively, under the moderate RCP 4.5 scenario (Fig. 3c, e). Across kingdoms, i.e. archaea, bacteria and fungi, the changes in alpha diversity and composition followed similar trends, but significant differences in their magnitude (Additional file 1: Figure S12). Fungal functional genes showed the highest projected increase in alpha diversity, while bacteria accounted for the greatest changes in composition (Additional file 1: Figure S12). Such a finding indicates cross-kingdom variations in the magnitude of functional diversity response to climate change, which constitutes a significant step beyond previous reports on taxonomic approaches which show distinct biogeographical patterns and responses to nutrient availability for archaea, bacteria and fungi [50, 54]. Interestingly, the projected changes showed highly congruent trends across functional gene families (Fig. 3d, f). These results are consistent with the rapid response documented for macro- and microbial genes under climate change experiments [12, 55] and suggest that temperature and precipitation changes may strongly influence functional gene assemblages mediating multiple ecosystem processes, likely producing biogeochemical feedbacks to the climate [56, 57].

Across Eurasia, the largest increase in alpha diversity will occur in regions North of 60° N, while assemblage composition will mainly shift between 45° and 75° N. These projections show for the first time the highest sensitivity of northern regions to functional diversity changes affecting multiple stream processes, such as biogeochemical cycling. Despite no previous studies have anticipated the future trends of microbial functional diversity, neither in terrestrial nor freshwater ecosystems, there are interesting reports about plant traits and certain ecosystem functions [58, 59]. For instance, temperature sensitivity of soil respiration is predicted to mainly increase with warming at high latitudes by affecting microbial assemblage compositions [59].

The predicted changes can be further visualized through the lens of key biogeochemical processes mediated by gene families that showed a consistent alpha diversity response to elevation. Such a response was especially evident for gene families associated with the decomposition of recalcitrant C, such as chitin, aromatics and lignin (Additional file 1: Figure S13). This finding suggests an increase in CO_2_ emissions through the stimulation of old recalcitrant C upstream and northward, likely producing a positive feedback to global warming [12]. In addition, all gene families linked to denitrification exhibited a similar alpha diversity response to elevation (Additional file 1: Figure S13). This finding suggests accelerated rates of nitrification with global warming that could increase anthropogenic emissions of N_2_O, a potent greenhouse gas contributing to climate change and stratospheric ozone destruction [60]. Other gene families associated with essential processes, such as nitrogen fixation, assimilatory nitrogen reduction and nitrification, also showed a consistent decline in alpha diversity upstream, therefore suggesting a high sensitivity of these processes towards climate change.

Although we here show a big picture on the large-scale functional implications of microbial diversity changes, two major caveats could be acknowledged regarding the spatial modelling framework. First, our projections comprise considerable extrapolated areas due to the relatively limited climate gradient covered by the studied mountains. Further studies are encouraged to broaden the climate envelope by including additional elevational gradients. Second, our modelling informs on the variance in functional gene alpha diversity and assemblage composition exclusively linked to climatic predictors while it does not consider local- and landscape-level predictors. The reasons behind this strategy are mainly connected to the primary role of climate in driving large-scale microbial functional diversity as shown by our study and previous reports [9, 10] and also to the availability of future scenarios for climate but not for local variables. In this sense, the employed climate data set is the best one currently available for large-spatial scales and remote areas where there is no locally measured climatic data accessible. Despite our approach shows clear trends of increasing diversity and compositional change under future climate scenarios, there are uncertainties related to the exact magnitude of such changes which should be adjusted when more sophisticated current and future climate data become available [61, 62]. This recommendation is especially applicable to compositional change because of the uncertainty associated with interpolated precipitation provided by the climate gridded dataset [63]. We note, however, that local physicochemical predictors vary within and among streams due to geological origins, climate variations, hydrological factors or anthropogenic disturbances, and have been demonstrated, together with landscape position, of substantial importance for microbial taxonomic diversity [64–66]. Although the inclusion of key local predictors will surely increase the accuracy of biodiversity projections under future climate scenarios, it currently constitutes a great challenge for large-scale studies. By assessing climate change effects on biodiversity from elevational gradients to large geographical extents and bearing in mind that the conclusions of our approach are subjected to the mentioned caveats, we here present a novel spatial exercise based on “elevation-for-latitude” substitution to provide the first general overview of climate change impacts on microbial functional diversity at the Eurasian scale.

## Conclusions

In summary, our results demonstrate how climate constrains microbial functional diversity in lotic fresh waters and contribute to a better understanding of ecosystem responses to climate change. Warming and altered precipitation regimes under future climate will affect alpha diversity and lead to compositional turnover, these phenomena being especially evident in mid- and high-latitude regions across Eurasia. The predicted changes indicate a functional response of microorganisms linked to crucial biogeochemical processes, such as nutrient cycling, organic matter decomposition and greenhouse gas emissions, and anticipate biogeochemical feedback to ongoing climate change. Taken together, our findings serve as a baseline for estimating climate change impacts on stream processes through the study of microbial functional gene assemblages. We finally recommend further tests based on metatranscriptomics, metaproteomics and metabolomics approaches to support the validity of such findings and encourage future research to include field observations covering wider climate gradients and manipulative experiments across ecosystems and biogeochemical cycles, especially focusing on fresh waters and other nutrients in addition to carbon.

## Methods

### Biological sampling

To span a wide climate gradient across Eurasia, we selected 52 stream sites located along three mountainsides from the subarctic, Mediterranean and subtropical regions (Additional file 1: Figure S1). For the subarctic region, we sampled the Bálggesvárri mountain in the Lyngen Alps Landscape Protected Area (Norway). This area presents a collection of glaciers and rocky peaks reaching a maximum elevation of 1833 m.a.s.l. For the Mediterranean region, samples were collected in Aigüestortes and Estany de Sant Maurici National Park. This area is known by its extensive set of glacial lakes located in the Pyrenees (Spain), a massive mountain range with its highest altitude reaching 3404 m.a.s.l. For the subtropical region, we selected the Laojun Mountain National Park, which is located at the junction of the Tibetan and Yungui Plateaus (China). This area presents a maximum elevation of 4513 m.a.s.l. and harbours typical mountain streams which, below 1800 m.a.s.l., turn into a deep, slowly discharging large river (Jinsha River, Upper Yangtze River). All these areas represent a landscape dominated by mixed evergreen and deciduous forests in lowlands, shrubs and grasses above the tree-line and bare rock peaks. Because landscape position has been reported of substantial importance for microbial taxonomic diversity [66–68], we selected for sampling only first, second at most, order streams to minimize the influence of such an additional confounding factor. Detailed information on the fieldwork procedures are described in Wang et al. [31]. In short, the sampling sites were evenly spaced according to elevation and ranged from 18 to 771 m.a.s.l. in Bálggesvárri (*n* = 17), from 850 to 2500 m.a.s.l. in the Pyrenees (*n* = 17), and from 1828 to 4045 m.a.s.l. in Laojun (*n* = 18). Fieldwork was carried out in Autumn 2009 in China and Summer and Autumn 2012 in Norway and Spain, respectively. We started the sampling from the highest elevation and ended in the lowest parts of the streams, where the elevation stopped varying substantially. Based on stream width, five to ten cross sections were established at each location. Along the transects, we randomly selected 10 stones from riffle/run habitats and collected biofilm subsamples from the surface of each stone by scraping off a 9-cm^2^ area using a sterilized sponge. Subsamples were then pooled into a composite sample for each site and immediately frozen at − 18 °C.

### Environmental data

We collected data on latitude, longitude and elevation as well as 11 spatial and physicochemical variables in situ or in the laboratory for each site (Additional file 1: Table S6) following the methods described in Wang et al. [31]. Briefly, the latitude, longitude and altitude were taken using a GPS unit. Shading (% of canopy cover) was estimated from 10 locations in evenly spaced perpendicular transects that covered the whole study stretch. The water depth, current velocity, width and substratum grain size were obtained from 10 random locations within the same transects. We also measured the water temperature, conductivity and pH at each site. In the laboratory, we analysed chlorophyll-a by the 90% acetone extraction method [69] as well as total nitrogen and total phosphorus by peroxodisulphate oxidation and the spectrophotometry method [70], respectively. For Bálggesvárri Mountain, total nitrogen data were not available because the concentration level did not reach the minimum detection threshold required for the method employed. Additionally, we extracted 19 climatic variables with a spatial resolution of 30 arc seconds (~ 1 km) from WorldClim v.1.4. (www.worldclim.org/current) for each site. This information is based on interpolated climate station data and represents the average conditions for years 1960–1990 [71] and constitutes the best climatic dataset currently available for large-spatial scales and remote zones where locally measured climatic data are not accessible.

### GeoChip procedures

We measured functional gene diversity and composition by applying GeoChip 4.0, a highly comprehensive (~ 82,000 probes covering 141,995 coding sequences) functional gene array widely used in biogeochemical, ecological and environmental analyses [7] because of its high optimization. GeoChip method presents several advantages compared to open-format technologies (e.g. metatranscriptomics, metaproteomics and metabolomics), such as high throughput, low detection limits, high reproducibility and potential for quantification, enabling a rapid performance with good correlations between target DNA concentrations and hybridization signal intensities [72]. Microbial DNA was extracted from the biofilm samples using the phenol chloroform method [73] and purified using the QIAquick Gel Extraction Kit (QIAGEN Sciences, Germantown, MD, USA). The purified DNA was then quantified with a PicoGreen Kit (Eugene, OR, USA) and used for the GeoChip 4.0 hybridization. The DNA from each sample (500 ng) was labelled with the fluorescent dye Cy-3 (GE Healthcare, California, USA) by random priming [74]. The DNA was purified as explained above and dried in a SpeedVac (Thermo Savant, New York, USA). Then, each dried and labelled DNA sample was resuspended in 42 μL of hybridization solution. This solution consisted of 1× HI-RPM hybridization buffer, 1× comparative genome hybridization blocking agent, 0.05 μg μL^−1^ Cot-1 DNA, 10 pM universal standard and 10% formamide (final concentrations). The solution was later denatured by remaining at 95 °C for 3 min. To remove the bubbles created during the denaturation process, the conditions were maintained at 37 °C for 30 min. The hybridizations were carried out at 67 °C for 24 h. Finally, the scanned images of the GeoChip hybridizations were obtained and converted by means of the Agilent Feature Extraction 11.5 software (Agilent Technologies, California, USA). For GeoChip data analyses, we employed signal intensity-based matrices as variation in functional gene abundances has an effect on diversity metrics and can also inform itself about environmental change [12]. The signal intensities were quantified and processed based on previous pipelines [74] as follows: (i) removing probes with a signal-to-noise ratio of less than 2.0; (ii) normalizing the signal intensity of each probe by dividing the total signal intensity of a sample and multiplying by a constant;( iii) removing singletons, i.e. genes detected only once on each mountain; and (iv) selecting those genes involved in the carbon (C), nitrogen (N), phosphorus (P) and sulphur (S) cycles and stress-related (St) processes.

### Mountain and continental scale patterns

Microbial functional genes are hierarchically organized, i.e. they are included in 88 functional gene families which are in turn grouped into five functional categories. Thus, alpha diversity and assemblage composition dissimilarity were calculated based on these two hierarchical levels. Firstly, we considered functional gene assemblages for the three kingdoms at the sampling site level which included all functional genes irrespective of their functional gene family and their functional category. Secondly, we repeated the procedure by grouping the functional genes into functional gene families. Then, we calculated the diversity and compositional dissimilarities of these functional gene assemblages. Given that various diversity metrics, such as observed richness, Chao1 estimated richness, Shannon-Wiener index, Simpson index and Inverse Simpson index, showed high correlations with each other (Pearson, *r* > 0.97 in all cases), we thus finally used alpha diversity based on the Shannon-Wiener index (base = exp(1)) because it is perhaps the most commonly employed diversity index in ecological research [75] and combines species richness (that is, functional gene richness in our case) and abundances (that is, signal intensities). For a more intuitive interpretation of diversity, we used true diversity, i.e. the effective number of attributes derived from the exponent-transformed Shannon entropy [76]. Cross-site and cross-mountain differences in functional gene compositions (referred as turnover [77] in our study) were evaluated by Bray-Curtis dissimilarity, which accounts for the variation in assemblage structure due to changes in identities and abundances. We considered this measure of beta diversity because we chose to use quantitative data (i.e. signal intensities) given the importance of abundance for ecosystem functioning [78] and to exclude joint absences as this is an appropriate strategy for analysing communities along environmental gradients [77]. We *log-*transformed signal intensities in all cases to emphasize the role of less-abundant genes as rare items can be essential in terms of ecosystem functioning [79]. Across mountains, we tested for overall and pairwise differences in mean alpha diversity by ANOVA and Bonferroni-corrected *t*-tests, respectively. Global and pairwise differences in assemblage composition were assessed by PERMANOVA (1000 permutations), with Bonferroni-adjusted *P* values in pairwise comparisons. Both analyses were performed on the overall functional gene pool as well as on functional genes grouped into kingdoms and functional categories (i.e. C, N, P, S and St). Within mountains, the elevational gradients in alpha diversity and composition were examined on functional gene assemblages grouped into kingdoms and functional gene families (grouped by functional categories). For better comparison of model coefficients, elevation was *z*-standardized. The relationships between alpha diversity and elevation were tested by LMs, and the significance was determined by F-statistics. For kingdoms, we tested models including linear and quadratic terms. When both models were significant (*P* < 0.05) and significantly different (ANOVA; *P* < 0.05), we selected as the best model the one that minimized the Akaike’s information criterion adjusted for small sample size (AICc). For gene families, we considered only linear relationships as the alpha diversity-elevation relationships were estimated through model slope values. The relationships between assemblage dissimilarity and elevational Euclidean distances were examined by LMs for both kingdoms and functional gene families, with their significance determined by the Mantel test (Pearson correlations, 1000 permutations). Pairwise comparisons of mean slopes from LMs assessing the alpha diversity and compositional turnover response to elevation across functional gene families were tested by Bonferroni-corrected *t*-tests. For this analysis, gene families were grouped into functional categories and pairwise differences checked firstly with and secondly without accounting for the mountain. Because spatial patterns are most likely weak for those gene families containing low richness of functional genes, they were classified into richness-based quartiles and those within the lower quartile were excluded from the analyses. Finally, we examined the elevational patterns for 88 gene families (their distribution across the 5 targeted functional categories can be seen at Additional file 1: Table S1).

### Pre-selection of climatic and local non-climatic predictors

To elucidate the drivers shaping the continental and mountain scale gradients in diversity and assemblage composition, we firstly selected ecologically relevant predictors that minimized collinearity to be included in the analyses. Prior to the initial selection, we ranked the individual influence of all predictors on alpha diversity and assemblage composition by RF analyses [80]. We then included in further analyses the best ranked predictors that were not strongly correlated with the others (Pearson; *r* < 0.7; Additional file 1: Figure S14) prioritizing those predictors with a clear influence on freshwater microbial diversity at high elevations [41], such as those related to the growing season conditions, i.e. linked to the kinetics of biological processes, and the winter precipitation, i.e. mediating the hydrology in mountains through snow accumulation. This procedure, as well as the analyses themselves (see below), were applied to each dataset, i.e. all sites and the sites from individual mountains, on the overall functional gene assemblages.

### Drivers underlying the biogeographical patterns

In order to get a comprehensive picture on how climatic and local non-climatic predictors contribute to the alpha diversity gradients, we employed an approach based on multiple statistical methods, including the HP, LMs and RFs. The combination of the HP and LMs provides robust evidence for meaningful predictors when both methods retain the same variables, and minimizes the probability of spurious results due to multicollinearity [39].

First, we used a multi-model approach to select the candidate model by running LMs on the full models (those containing all potential combinations of pre-selected single predictors) with alpha diversity as response variable. The models were ranked according to their AICc values and those with minimum AICc were chosen as candidate models. These models were further checked for spatial autocorrelation by Moran’s tests [81] and validated by visually checking their residuals for normality and homoscedasticity [82].

Second, we perform the HP, LM and RF analyses to evaluate the relationships between the predictors included in the candidate model and the response variable. For the HP analyses, the significance of the independent effects on the response variable was tested through a 1000 randomization-based procedure [39]. For LMs, we *z*-standardized the predictors in order to better compare the coefficients within models. For all RFs (see also above), we set 3000 as the number of trees, and the number of predictors used in each split was one third of the included predictors; those variables showing negative or zero importance values were considered not predictive [80]. For better comparison of the relative contributions of predictors, we transformed the variable importance scores into percentages relative to the sum of the importance values. To assess how predictors contributed to assemblage composition, we followed the same procedure employed for alpha diversity but using as a response variable the first axis coordinates from principal coordinate analysis (PCoA; Additional file 1: Figure S3). When assessing the cross-mountain gradient in composition, this procedure was further supported by GDMs, which predict spatial variation in assemblage composition between site pairs by modelling the response of biological dissimilarity matrices to environmental predictors. We run GDMs, plotted the I-splines for each predictor and obtained the impact of all individual predictors on the response dissimilarities (estimated as the variance explained by each predictor when the rest were kept constant). The uncertainty in the fitted I-splines was checked by plotting I-splines with error bands from 100 interactions-based bootstrapping [83], each data subsample retaining 70% of the sites from the full site-pair table. The significances of the full model and the individual predictors were assessed by a 100 permutation-based procedure [84]. For both the PCoA and GDMs, the biological distances were obtained by applying the Bray-Curtis dissimilarity on signal intensity-based matrices previously log-transformed.

Additionally, we divided the variance in alpha diversity and composition associated with the climatic and local non-climatic predictors by variation partitioning [85] based on linear regression for alpha diversity and redundancy analysis [86] for the compositional matrices. In the latter case, signal intensity-based matrices were previously Hellinger-transformed to meet the requirements of linear ordination methods [87]. The selection of predictors was performed by the ordiR2step procedure [88], which bases the forward selection of variables on adjusted *R*^2^ and *P* values of the initial pre-selected variables. When no predictors were retained for the local non-climatic set, the variance in alpha diversity in relation to the climatic predictors was checked by LMs, whereas the variance in composition was checked by a Mantel test.

### Projections under climate change scenarios

We considered future climate conditions based on the most “optimistic”, one intermediate and the most “pessimistic” expectations of greenhouse emission rate scenarios, that is, the representative concentration pathways (RCPs) 2.6, 4.5 and 8.5. They broadly equal to atmospheric CO_2_ concentrations of 490, 650 and 1,370 p.p.m., respectively, by the end of this century [53]. These future climate conditions based on the mentioned three CO_2_ emission scenarios come from the Fifth Assessment Report of The Intergovernmental Panel on Climate Change [25] and are available at the *WorldClim* site (www.worldclim.org/CMIP5v1) [71]. We downloaded the climatic variables from all individual downscaled and bias-corrected climate model outputs available (*n* = 15–19) in the database at 2.5 arc minute spatial resolutions. We only considered the data that represent future climatic conditions averaged over the period from 2061 to 2080. For subsequent analyses, we used the ensemble means of each climatic variable over the individual climate model outputs.

We projected the changes in alpha diversity and shifts in assemblage composition over the entire Eurasian domain at a spatial resolution of 2.5 arc minutes combining the TWQ and PCQ as predictor variables, i.e. the best predictors of alpha diversity and assemblage composition, respectively. The current and future alpha diversities were obtained using LMs based on linear relationships. The variation in functional gene composition was projected using GDMs [44, 89] with a “space-for-time” substitution [90] based on Bray-Curtis dissimilarity matrices that were previously log-transformed. GDMs were run on the whole functional gene pool and on functional gene subsets for each gene family. The projections were shown on fluvial systems by masking the outputs with the global river classification layer [91]. We excluded rivers that were classified as “very small” and “small” in the mask and applied a 5-km buffer around the streams for an adequate visualization of resulting continental-scale patterns.

All statistical analyses and plots were run in R statistical software V3.5.3 by using the packages vegan V2.54 [92], ggplot2 V3.1.1 [93], MuMIn V1.43.6 [94], hier.part V1.0-4 [95], gdm V1.3.11 [84], randomForestSRC V2.9.0 [96], raster V2.8-19 [97] and corrplot [98].

## Supplementary information

**Additional file 1.**

## Data Availability

The GeoChip 4.0 dataset generated during the current study is available in the Gene Expression Omnibus repository with the accession number GSE128826, [www.ncbi.nlm.nih.gov/geo/]. Any other relevant data for this study are available from the corresponding author on reasonable request.
