# Supplementary material for: Climate mediates continental scale patterns of stream microbial functional diversity
Source: Microbiome. 2020 Jun 13;8:92. doi: 10.1186/s40168-020-00873-2 (PMC7293791; doi:10.1186/s40168-020-00873-2)
Supplement: Supplementary file 2 — Additional file 1. [file 40168_2020_873_MOESM1_ESM.docx]

**Supplementary materials for the manuscript:**

**Climate mediates continental scale patterns of stream microbial functional diversity**

Félix Picazo, Annika Vilmi, Juha Aalto, Janne Soininen, Emilio O. Casamayor, Yongqin Liu, Qinglong Wu, Lijuan Ren, Jizhong Zhou, Ji Shen, Jianjun Wang

**This file includes:**

**Tables S1 to S6**

**Figures S1 to S14**

Table S1. Richness of microbial functional genes associated with the cycling of carbon, nitrogen, phosphorus and sulphur as well as stress-related processes across the whole study extent, individual mountains, kingdoms and functional gene categories. In brackets, the number of functional gene families included within each functional gene category.

|  |  | **Eurasia** |  | **Norway** |  | **Spain** |  | **China** |
| --- | --- | --- | --- | --- | --- | --- | --- | --- |
| **All genes** |  | 15289 |  | 10257 |  | 10555 |  | 12646 |
| **Archaea** |  | 538 |  | 357 |  | 380 |  | 430 |
| **Bacteria** |  | 13780 |  | 9253 |  | 9474 |  | 11390 |
| **Fungi** |  | 885 |  | 599 |  | 642 |  | 760 |
| **Carbon (32)** |  | 3916 |  | 2672 |  | 2733 |  | 3259 |
| **Nitrogen (13)** |  | 2681 |  | 1783 |  | 1881 |  | 2185 |
| **Phosphorus (3)** |  | 478 |  | 325 |  | 334 |  | 406 |
| **Sulphur (9)** |  | 1469 |  | 921 |  | 999 |  | 1225 |
| **Stress (31)** |  | 6745 |  | 4556 |  | 4608 |  | 5571 |

Table S2. Alpha diversity-elevation and compositional turnover-elevational distance relationships. For alpha diversity (calculated as Shannon-Wiener index), model coefficients, *P* values and adjusted *R*^2^ from linear models (LMs) are shown. For compositional turnover (based on Bray-Curtis dissimilarity), model coefficients from LMs, and *P* and correlation values from Mantel tests (1,000 permutations) are shown. Coef, model coefficients; Elev. Dist, elevational distance.

| **Alpha diversity** |  |  |  |  |  |  | **Coef** |  | ***P*** |  | **Adjusted *R*^2^** |
| --- | --- | --- | --- | --- | --- | --- | --- | --- | --- | --- | --- |
|  | **Norway** |  | **Archaea** |  | **Intercept** |  | 193.9 |  | < 0.001 |  | 0.353 |
|  |  |  |  |  | **Elevation** |  | -18.1 |  | 0.007 |  |  |
|  |  |  | **Bacteria** |  | **Intercept** |  | 4717.0 |  | < 0.001 |  | 0.302 |
|  |  |  |  |  | **Elevation** |  | -606.0 |  | 0.013 |  |  |
|  |  |  | **Fungi** |  | **Intercept** |  | 299.3 |  | < 0.001 |  | 0.389 |
|  |  |  |  |  | **Elevation** |  | -48.2 |  | 0.004 |  |  |
|  | **Spain** |  | **Archaea** |  | **Intercept** |  | 200.1 |  | < 0.001 |  | 0.126 |
|  |  |  |  |  | **Elevation** |  | -7.2 |  | 0.089 |  |  |
|  |  |  | **Bacteria** |  | **Intercept** |  | 4699.2 |  | < 0.001 |  | 0.354 |
|  |  |  |  |  | **Elevation** |  | -476.9 |  | 0.007 |  |  |
|  |  |  | **Fungi** |  | **Intercept** |  | 306.4 |  | < 0.001 |  | 0.303 |
|  |  |  |  |  | **Elevation** |  | -35.2 |  | 0.013 |  |  |
|  | **China** |  | **Archaea** |  | **Intercept** |  | 222.4 |  | < 0.001 |  | 0.652 |
|  |  |  |  |  | **Elevation** |  | -25.4 |  | < 0.001 |  |  |
|  |  |  | **Bacteria** |  | **Intercept** |  | 5560.8 |  | < 0.001 |  | 0.806 |
|  |  |  |  |  | **Elevation** |  | -1155.4 |  | < 0.001 |  |  |
|  |  |  |  |  | **Elevation^2^** |  | 349.4 |  | 0.015 |  |  |
|  |  |  | **Fungi** |  | **Intercept** |  | 355.2 |  | < 0.001 |  | 0.824 |
|  |  |  |  |  | **Elevation** |  | -105.2 |  | < 0.001 |  |  |
|  |  |  |  |  | **Elevation^2^** |  | 31.5 |  | 0.011 |  |  |
| **Assemblage composition** |  |  |  |  |  |  | **Coef** |  | ***P*** |  | ***r*-cor** |
|  | **Norway** |  | **Archaea** |  | **Intercept** |  | 0.254 |  | 0.043 |  | 0.184 |
|  |  |  |  |  | **Elev. Dist.** |  | 0.011 |  |  |  |  |
|  |  |  | **Bacteria** |  | **Intercept** |  | 0.272 |  | 0.030 |  | 0.194 |
|  |  |  |  |  | **Elev. Dist.** |  | 0.014 |  |  |  |  |
|  |  |  | **Fungi** |  | **Intercept** |  | 0.297 |  | 0.015 |  | 0.239 |
|  |  |  |  |  | **Elev. Dist.** |  | 0.019 |  |  |  |  |
|  | **Spain** |  | **Archaea** |  | **Intercept** |  | 0.272 |  | 0.005 |  | 0.383 |
|  |  |  |  |  | **Elev. Dist.** |  | 0.021 |  |  |  |  |
|  |  |  | **Bacteria** |  | **Intercept** |  | 0.283 |  | 0.017 |  | 0.289 |
|  |  |  |  |  | **Elev. Dist.** |  | 0.020 |  |  |  |  |
|  |  |  | **Fungi** |  | **Intercept** |  | 0.322 |  | 0.033 |  | 0.241 |
|  |  |  |  |  | **Elev. Dist.** |  | 0.017 |  |  |  |  |
|  | **China** |  | **Archaea** |  | **Intercept** |  | 0.221 |  | 0.001 |  | 0.650 |
|  |  |  |  |  | **Elev. Dist.** |  | 0.063 |  |  |  |  |
|  |  |  | **Bacteria** |  | **Intercept** |  | 0.217 |  | 0.001 |  | 0.705 |
|  |  |  |  |  | **Elev. Dist.** |  | 0.071 |  |  |  |  |
|  |  |  | **Fungi** |  | **Intercept** |  | 0.234 |  | 0.001 |  | 0.692 |
|  |  |  |  |  | **Elev. Dist.** |  | 0.084 |  |  |  |  |

Table S3. Cross-mountain differences in microbial functional gene diversity for the overall gene pool and genes grouped into kingdoms and functional gene categories. Differences in mean alpha diversity (Shannon-Wiener index) and assemblage composition (Bray-Curtis dissimilarity) were tested by ANOVA and PERMANOVA analyses, respectively. Post hoc pairwise comparisons for alpha diversity and assemblage composition were based on Bonferroni-corrected *t*-tests and PERMANOVA analyses with Bonferroni-adjusted *P* values, respectively. The letters for post hoc analyses on alpha diversity indicate that the differences are significant (*P* < 0.05). NO, Norway; SP, Spain; CH, China.

| **Alpha diversity** |  |  | **F statistic Df (2, 49)** |  | ***P*** |  | **Mean values NO; SP; CH** |  | **Post hoc NO; SP; CH** |
| --- | --- | --- | --- | --- | --- | --- | --- | --- | --- |
|  | **All genes** |  | 7.754 |  | 0.001 |  | 5233.6; 5231.7; 6525.3 |  | a; a; b |
|  | **Archaea** |  | 5.650 |  | 0.006 |  | 193.9; 200.1; 222.4 |  | a; a; b |
|  | **Bacteria** |  | 8.011 |  | < 0.001 |  | 4717.0; 4699.2; 5890.8 |  | a; a; b |
|  | **Fungi** |  | 5.747 |  | 0.006 |  | 299.3; 306.4; 385.0 |  | a; a; b |
|  | **Carbon** |  | 6.975 |  | 0.002 |  | 1387.0; 1378.3; 1706.0 |  | a; a; b |
|  | **Nitrogen** |  | 8.415 |  | < 0.001 |  | 885.1; 910.1; 1123.4 |  | a; a; b |
|  | **Phosphorus** |  | 7.026 |  | 0.002 |  | 178.7; 178.8; 219.8 |  | a; a; b |
|  | **Sulphur** |  | 7.488 |  | 0.001 |  | 448.6; 472.8; 600.3 |  | a; a; b |
|  | **Stress** |  | 8.054 |  | < 0.001 |  | 2334.1; 2291.7; 2875.8 |  | a; a; b |
| **Aseemblage composition** |  |  | **Global *R*^2^** |  | ***P*** |  | **Pairwise *R*^2^ NO&SP; NO&CH; SP&CH** |  | **Pairwise *P*  NO&SP; NO&CH; SP&CH** |
|  | **All genes** |  | 0.387 |  | 0.001 |  | 0.096; 0.382; 0.400 |  | 0.003; 0.003; 0.003 |
|  | **Archaea** |  | 0.436 |  | 0.001 |  | 0.136; 0.431; 0.436 |  | 0.003; 0.003; 0.003 |
|  | **Bacteria** |  | 0.390 |  | 0.001 |  | 0.095; 0.385; 0.404 |  | 0.012; 0.003; 0.003 |
|  | **Fungi** |  | 0.309 |  | 0.001 |  | 0.078; 0.302; 0.319 |  | 0.021; 0.003; 0.003 |
|  | **Carbon** |  | 0.383 |  | 0.001 |  | 0.092; 0.376; 0.399 |  | 0.003; 0.003; 0.003 |
|  | **Nitrogen** |  | 0.394 |  | 0.001 |  | 0.105; 0.391; 0.407 |  | 0.006; 0.003; 0.003 |
|  | **Phosphorus** |  | 0.375 |  | 0.001 |  | 0.090; 0.363; 0.390 |  | 0.006; 0.003; 0.003 |
|  | **Sulphur** |  | 0.360 |  | 0.001 |  | 0.092; 0.360; 0.360 |  | 0.012; 0.003; 0.003 |
|  | **Stress** |  | 0.393 |  | 0.001 |  | 0.095; 0.386; 0.407 |  | 0.006; 0.003; 0.003 |

Table S4. Response of alpha diversity (Shannon-Wiener index) and assemblage composition to climatic and local non-climatic predictors from linear models (LMs) across the whole study extent and individual mountains. Assemblage composition was approached from the first axis of principal coordinate analysis (Bray-Curtis dissimilarity). Model coefficients, *P* values and adjusted *R*^2^ are shown. Coef, model coefficients; TWQ, mean temperature of the warmest quarter; TAP, annual precipitation; Chl-a, chlorophyll-*a*; Cveloc, current velocity; PCQ, precipitation of the coldest quarter; TN, total nitrogen; IST, isothermality; TSE, temperature seasonality; TAR, temperature annual range.

| **Alpha diversity** |  |  |  |  | **Coef** |  | ***P*** |  | **Adjusted *R*^2^** |
| --- | --- | --- | --- | --- | --- | --- | --- | --- | --- |
|  | **Eurasia** |  | **Depth** |  | -0.274 |  | 0.006 |  | 0.645 |
|  |  |  | **TAP** |  | -0.180 |  | 0.066 |  |  |
|  |  |  | **TWQ** |  | 0.914 |  | < 0.001 |  |  |
|  | **Norway** |  | **Chl-a** |  | 0.494 |  | 0.016 |  | 0.603 |
|  |  |  | **Cveloc** |  | 0.395 |  | 0.076 |  |  |
|  |  |  | **TWQ** |  | 1.120 |  | < 0.001 |  |  |
|  | **Spain** |  | **Depth** |  | -0.516 |  | 0.017 |  | 0.483 |
|  |  |  | **TWQ** |  | 0.720 |  | 0.002 |  |  |
|  | **China** |  | **Cveloc** |  | -0.343 |  | 0.004 |  | 0.828 |
|  |  |  | **TWQ** |  | 0.930 |  | < 0.001 |  |  |
| **Assemblage composition** |  |  |  |  | **Coef** |  | ***P*** |  | **Adjusted *R*^2^** |
|  | **Eurasia** |  | **Depth** |  | -0.143 |  | 0.005 |  | 0.918 |
|  |  |  | **PCQ** |  | -0.858 |  | < 0.001 |  |  |
|  |  |  | **Shading** |  | 0.081 |  | 0.066 |  |  |
|  |  |  | **TAP** |  | 0.377 |  | < 0.001 |  |  |
|  |  |  | **TWQ** |  | 0.240 |  | < 0.001 |  |  |
|  | **Norway** |  | **Chl-a** |  | 0.423 |  | 0.038 |  | 0.569 |
|  |  |  | **TWQ** |  | 0.887 |  | < 0.001 |  |  |
|  | **Spain** |  | **Depth** |  | -0.400 |  | 0.060 |  | 0.453 |
|  |  |  | **TWQ** |  | 0.742 |  | 0.002 |  |  |
|  | **China** |  | **IST** |  | 0.315 |  | 0.039 |  | 0.839 |
|  |  |  | **TAR** |  | 0.839 |  | < 0.001 |  |  |
|  |  |  | **TN** |  | -0.247 |  | 0.028 |  |  |
|  |  |  | **TSE** |  | -0.519 |  | 0.003 |  |  |

**Table S5.** Results of the models examining the effect of temperature of warmest quarter (TWQ), precipitation of the coldest quarter (PCQ), conductivity (cond), latitude (lat) and their interactions (TWQ × lat, PCQ × lat, cond × lat) on alpha diversity and assemblage composition. Alpha diversity was calculated as the Shannon-Wiener index and assemblage composition was approached from the first axis of principal coordinate analysis (Bray-Curtis dissimilarity). Global model significance (*P*), adjusted *R*^2^ (Adj. *R*^2^), Akaike weight (weight), predictor effect sizes and their significance are shown for each model (significant terms are denoted by **P* < 0.05, ***P* < 0.01, *P**** < 0.001).

| **Alpha diversity** |  | **TWQ** |  | **cond** |  | **lat** |  | **TWQ × lat** |  | **cond × lat** |  | ***P*** |  | **Adj. *R*^2^** |  | **weight** |
| --- | --- | --- | --- | --- | --- | --- | --- | --- | --- | --- | --- | --- | --- | --- | --- | --- |
|  |  | 0.925*** |  |  |  | 0.281 |  | -0.085 |  |  |  | < 0.001 |  | 0.578 |  | 0.721 |
|  |  | 0.739*** |  |  |  |  |  |  |  |  |  | < 0.001 |  | 0.537 |  | 0.251 |
|  |  |  |  | 0.710*** |  |  |  |  |  |  |  | < 0.001 |  | 0.495 |  | 0.025 |
|  |  |  |  | 0.552 |  | −0.094 |  |  |  | −0.151 |  | < 0.001 |  | 0.476 |  | 0.003 |
|  |  |  |  |  |  | −0.386** |  |  |  |  |  | 0.005 |  | 0.132 |  | 0.000 |
| **Assemblage composition** |  | **PCQ** |  | **cond** |  | **lat** |  | **PCQ × lat** |  | **cond × lat** |  | ***P*** |  | **Adj. *R*^2^** |  | **weight** |
|  |  | −1.029*** |  |  |  | −0.647*** |  | −0.703 |  |  |  | < 0.001 |  | 0.896 |  | 1.000 |
|  |  | −0.845*** |  |  |  |  |  |  |  |  |  | < 0.001 |  | 0.709 |  | 0.000 |
|  |  |  |  | −0.414 |  | −0.951*** |  |  |  | −0.743* |  | < 0.001 |  | 0.609 |  | 0.000 |
|  |  |  |  |  |  | −0.708*** |  |  |  |  |  | < 0.001 |  | 0.491 |  | 0.000 |
|  |  |  |  | 0.623*** |  |  |  |  |  |  |  | < 0.001 |  | 0.376 |  | 0.000 |

**Table S6.** Data summary regarding elevation, the two climatic predictors used to assess the changes in diversity and composition under future climate scenarios (average from period 1960-1990) as well as the 11 local non-climatic variables measured *in situ* or in the lab across the study sites. We show the mean, standard deviation and range for each predictor within each mountain. Across-mountain differences in mean values were tested by ANOVA analyses. The letters following the mean values indicate significant (*P* < 0.05) differences assessed by Bonferroni-corrected pairwise *t*-test post hoc analyses. n, number of sampling sites; SD, standard deviation; Chl-a, chlorophyll-*a*; Cveloc, current velocity; Cond, conductivity; TP, total phosphorus; TN, total nitrogen. NA denotes that total nitrogen data were not available because the concentration level did not reach the minimum detection threshold required for the method employed. ****P* < 0.001; ***P* < 0.01; **P* < 0.05; n.s., non-significant.

| **ANOVA** | ***P*** | *** | *** | *** | *** | ** | *** | *** | n.s. | *** | *** | *** | n.s. | ** | ** |
| --- | --- | --- | --- | --- | --- | --- | --- | --- | --- | --- | --- | --- | --- | --- | --- |
|  |  |  |  |  |  |  |  |  |  |  |  |  |  |  |  |
|  | **F** | 104.5 | 31.4 | 471.0 | 11.0 | 6.4 | 9.4 | 20.7 | 0.1 | 31.5 | 51.3 | 12.9 | 1.7 | 7.7 | 6.4 |
|  |  |  |  |  |  |  |  |  |  |  |  |  |  |  |  |
| **China (n = 18)** | **range** | 1828-4045 | 9.2-20.9 | 25.0-46.0 | 1.52-40.36 | 1.0-50.0 | 8.0-50.0 | 2.25-5.10 | 0-93 | 0.1-1.0 | 6.3-8.9 | 140.0-1870.0 | 0.08-2.48 | 8.32-33.43 | 0.0-13.3 |
|  |  |  |  |  |  |  |  |  |  |  |  |  |  |  |  |
|  | **SD** | 641 | 3.5 | 5.5 | 11.14 | 12.7 | 10.8 | 0.92 | 30 | 0.2 | 0.8 | 557.8 | 0.59 | 6.89 | 4.2 |
|  |  |  |  |  |  |  |  |  |  |  |  |  |  |  |  |
|  | **mean** | 2712 c | 16.0 c | 32.2 c | 11.83 b | 11.5 b | 27.3 b | 3.61 b | 22 a | 0.7 c | 7.5 b | 621.1 b | 0.47 a | 18.67 b | 5.9 a |
|  |  |  |  |  |  |  |  |  |  |  |  |  |  |  |  |
| **Spain (n = 17)** | **range** | 850-2500 | 8.3-16.0 | 214.0-346.0 | 2.50-15.76 | 0.6-10.0 | 7.5-56.7 | 1.68-4.23 | 0-80 | 0.0-0.4 | 6.8-8.2 | 15.5-856.0 | 0.20-0.49 | 9.86-19.25 | 6.3-13.0 |
|  |  |  |  |  |  |  |  |  |  |  |  |  |  |  |  |
|  | **SD** | 472 | 2.3 | 39.7 | 4.11 | 3.0 | 14.0 | 0.56 | 24 | 0.1 | 0.4 | 209.0 | 0.06 | 2.76 | 2.2 |
|  |  |  |  |  |  |  |  |  |  |  |  |  |  |  |  |
|  | **mean** | 1671 b | 12.3 b | 271.8 b | 7.78 b | 4.8 a | 26.6 b | 3.28 b | 20 a | 0.2 a | 7.5 b | 189.5 a | 0.26 a | 13.70 a | 9.6 b |
|  |  |  |  |  |  |  |  |  |  |  |  |  |  |  |  |
| **Norway (n = 17)** | **range** | 18-771 | 7.2-10.8 | 145.0-157.0 | 0.07-2.17 | 0.9-8.0 | 8.5-21.4 | 1.65-2.63 | 0-66 | 0.2-0.8 | 5.2-6.2 | 15.3-85.7 | 0.10-0.46 | NA | 1.7-9.8 |
|  |  |  |  |  |  |  |  |  |  |  |  |  |  |  |  |
|  | **SD** | 247 | 1.3 | 4.3 | 0.627 | 2.2 | 3.5 | 0.293 | 21 | 0.2 | 0.3 | 29.0 | 0.114 | NA | 2.7 |
|  |  |  |  |  |  |  |  |  |  |  |  |  |  |  |  |
|  | **mean** | 343 a | 9.1 a | 149.9 a | 0.86 a | 2.4 a | 13.6 a | 2.24 a | 18 a | 0.5 b | 5.7 a | 44.3 a | 0.29 a | NA | 7.0 a |
|  |  |  |  |  |  |  |  |  |  |  |  |  |  |  |  |
|  |  | **Elevation (m)** | **TWQ (ºC)** | **PCQ (mm)** | **Chl-a (µg cm^-2^)** | **Width (m)** | **Depth (cm)** | **Ssize (cm)** | **Shading (%)** | **Cveloc (m s^-1^)** | **pH** | **Cond (µS cm^-1^)** | **TP (ppm)** | **TN (ppm)** | **Water T (ºC)** |


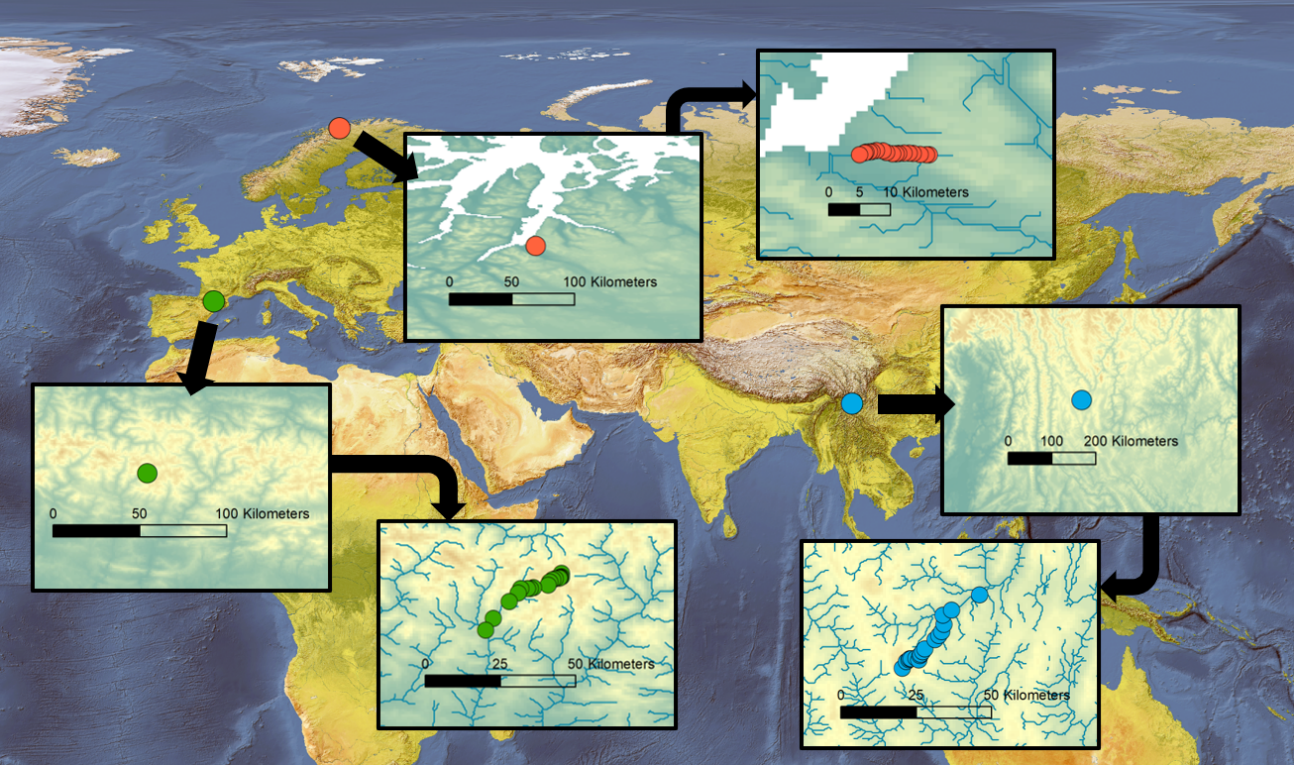


Figure S1. Study area showing the 52 sampled stream sites, the three studied mountainsides and their location across different climatic regions of Eurasia. For the subarctic region, we sampled the Bálggesvárri mountain in Norway (red). For the Mediterranean region, samples were collected in the Pyrenees mountains in Spain (green). For the subtropical region, we selected the Laojun Mountain National Park in China (blue). The sampling sites were evenly spaced according to elevation and ranged from 18 to 771 m.a.s.l. in Bálggesvárri (n = 17), from 850 to 2,500 m.a.s.l. in Pyrenees (n = 17), and from 1,828 to 4,045 m.a.s.l. in Laojun (n = 18). Sources: 1:10m resolution layer from Natural Earth project (<https://www.naturalearthdata.com/downloads/10m-raster-data/10m-natural-earth-1/>), GTOPO30 layer at 30 arc-sencond resolution (<https://www.usgs.gov/centers/eros/science>) and GloRic version 1.0 layer at 15 arc-second resolution (<https://www.hydrosheds.org/page/gloric>).


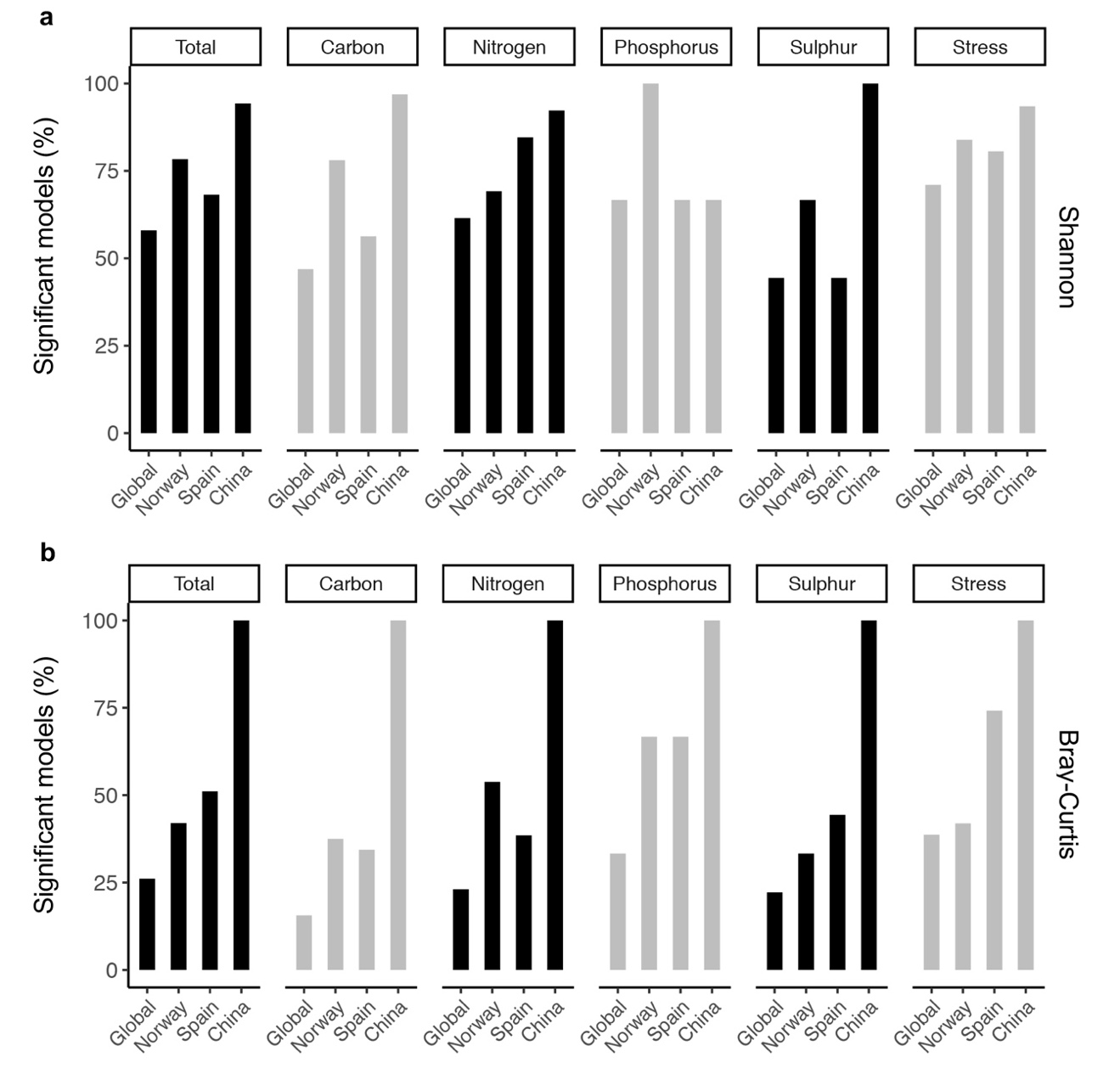


Figure S2. Proportion of functional gene families showing significant (*P* < 0.05) elevational patterns for alpha diversity (a) and compositional turnover (b), calculated over the overall gene pool and functional genes grouped into functional categories. The relationships between alpha diversity (Shannon-Wiener index) and elevation were examined by linear models (LMs), and the model significances (*P* < 0.05) were determined with F-statistics. The relationships between compositional turnover (Bray-Curtis dissimilarity) and elevational distance were calculated by LMs, and the model significances (*P* < 0.05) were obtained by Mantel tests. Only families within the first, second and third quartiles of functional gene richness were considered.


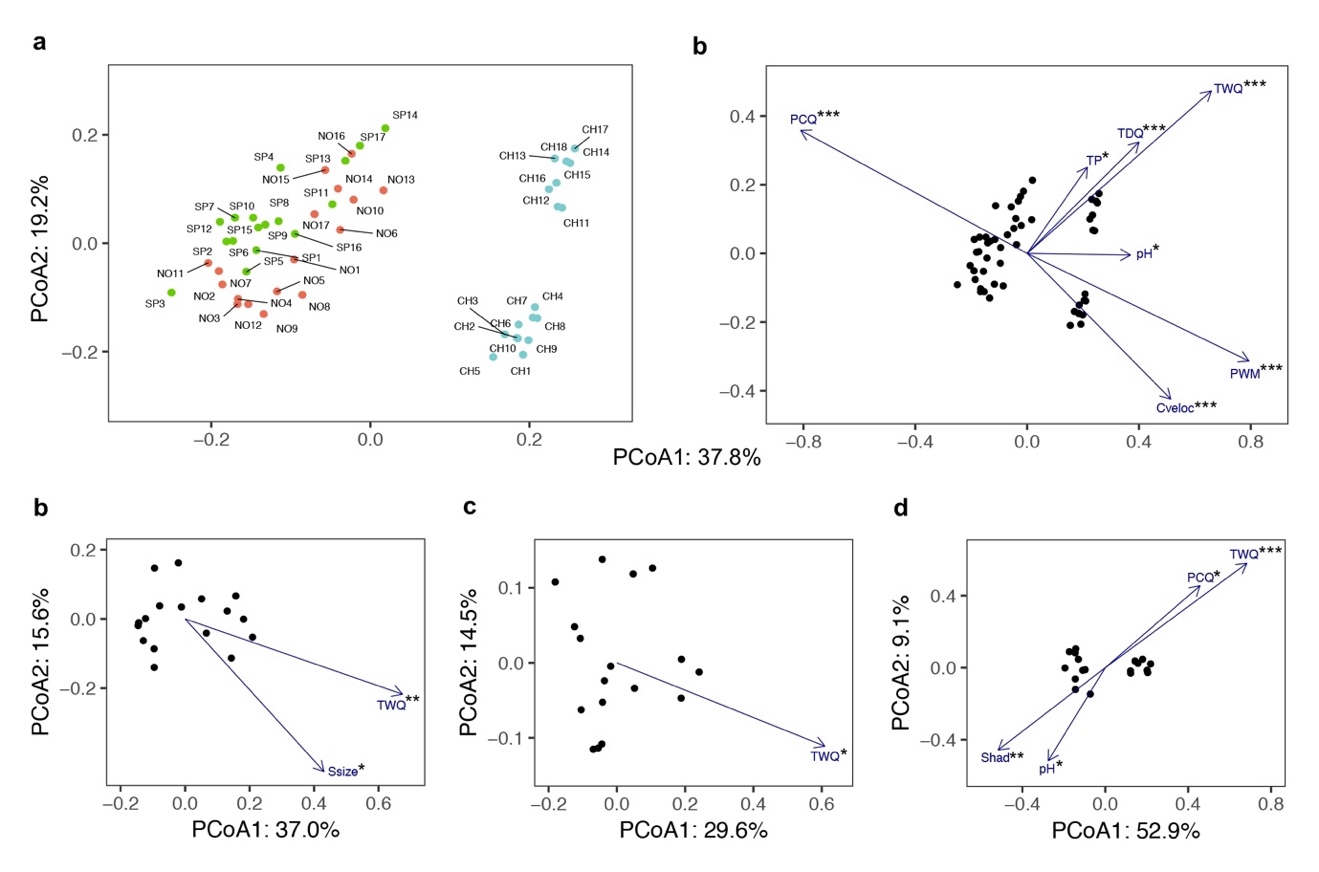


Figure S3. Principal coordinate analyses (PCoA; Bray Curtis dissimilarity) and environmental fitting on the overall functional gene pool for the whole study extent (a, b), and the mountains in Norway (c), Spain (d) and China (e). For the environmental fitting, all pre-selected variables for each dataset were included (see methods and Figure S13). Only significant predictors are shown (**P* < 0.05; ***P* < 0.01; ****P* < 0.001). Site numbers in (a) are ordered according to elevation from high to low altitudes. NO, Norway; SP, Spain; CH, China; PCQ, precipitation of the coldest quarter; TDQ, mean temperature of the driest quarter; TWQ, mean temperature of the warmest quarter; PWM, precipitation of the wettest month; TP, total phosphorus; Cveloc, current velocity; Shad, shading.


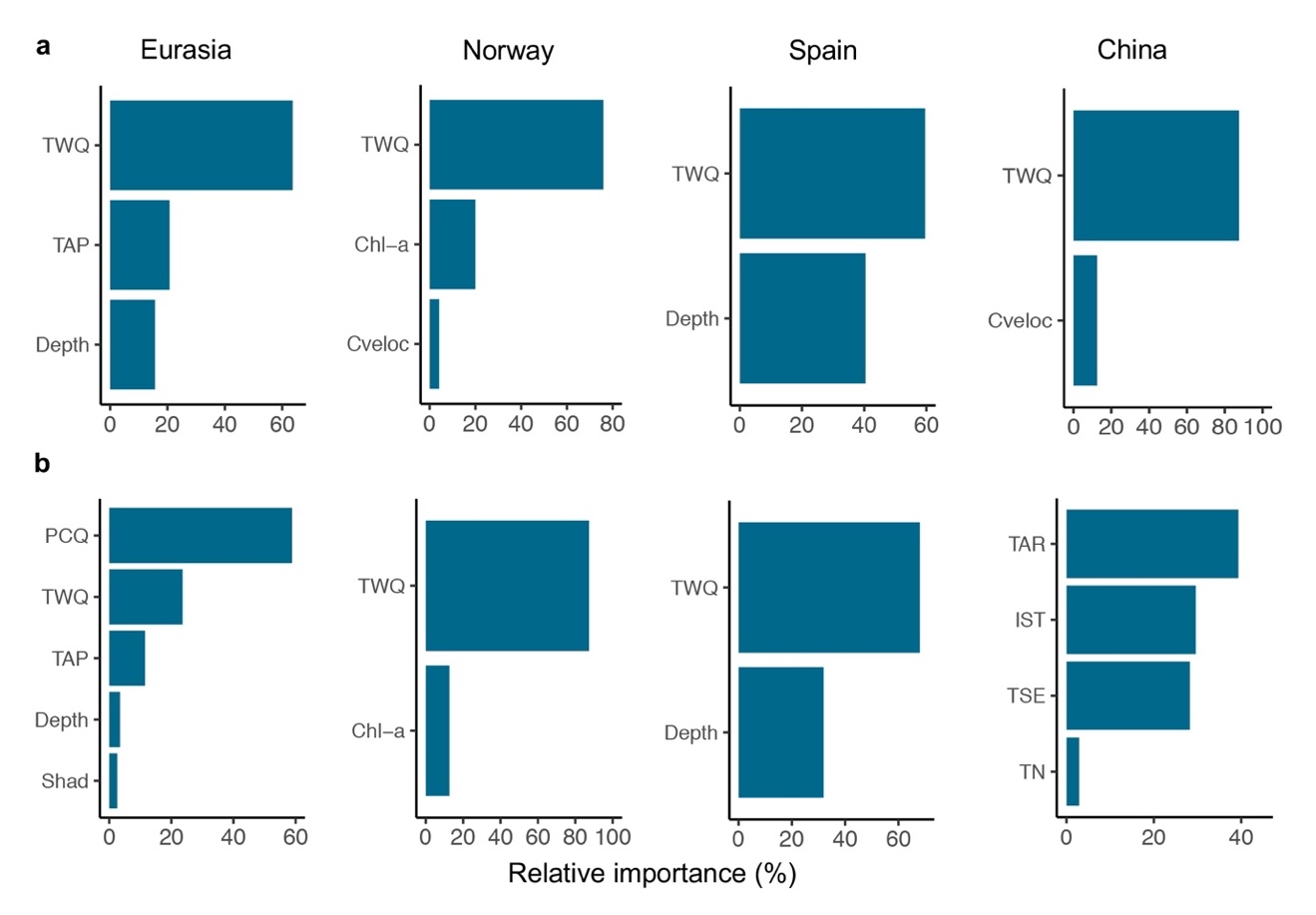


Figure S4. Relative importance of predictors in explaining overall alpha diversity (a) and assemblage composition (b) on the whole study extent and individual mountains, assessed by random forest analyses. Alpha diversity was calculated as the Shannon-Wiener index and assemblage composition was approached from the first axis of principal coordinate analysis (Bray-Curtis dissimilarity). TWQ, mean temperature of the warmest quarter; TAP, total annual precipitation; PCQ, precipitation of the coldest quarter; TAR, temperature annual range; TSE, temperature seasonality; IST, isothermality; Chl-a, chlorophyll-*a*; Cveloc, current velocity; Shad, shading; TN, total nitrogen.


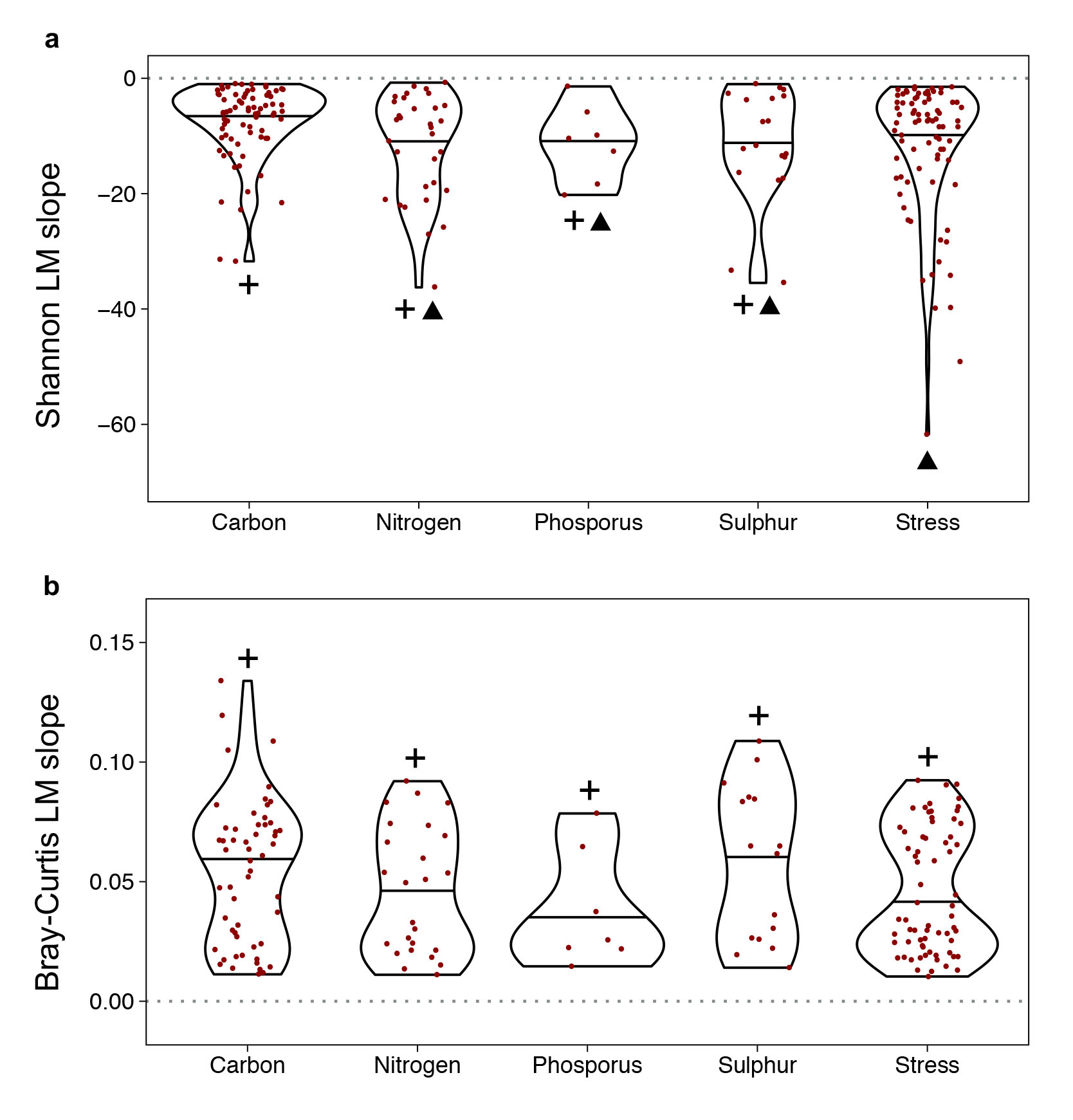


Figure S5. Slopes of linear models on the alpha diversity-elevation (a) and compositional turnover-elevational distance (b) relationships for gene families. Slope values were grouped according to functional categories without considering the mountain. The relationships between alpha diversity (Shannon-Wiener index) and elevation were examined by linear models (LMs), and the model significances were determined with F-statistics (*P* < 0.05). The relationships between compositional turnover (Bray-Curtis dissimilarity) and elevational distance were calculated by LMs, and the model significances (*P* < 0.05) were obtained by a Mantel test. Pairwise comparisons for alpha diversity and compositional turnover were based on Bonferroni-corrected *t*-tests and PERMANOVA analyses with Bonferroni-adjusted *P* values, respectively. The symbols + and ▲ indicate that the differences are significant (*P* < 0.05). The violin boxplots depict the median and the first and the third quartiles of significant (*P* < 0.05) models slopes based on functional gene families. Only families within the first, second and third quartiles of functional gene richness were considered.


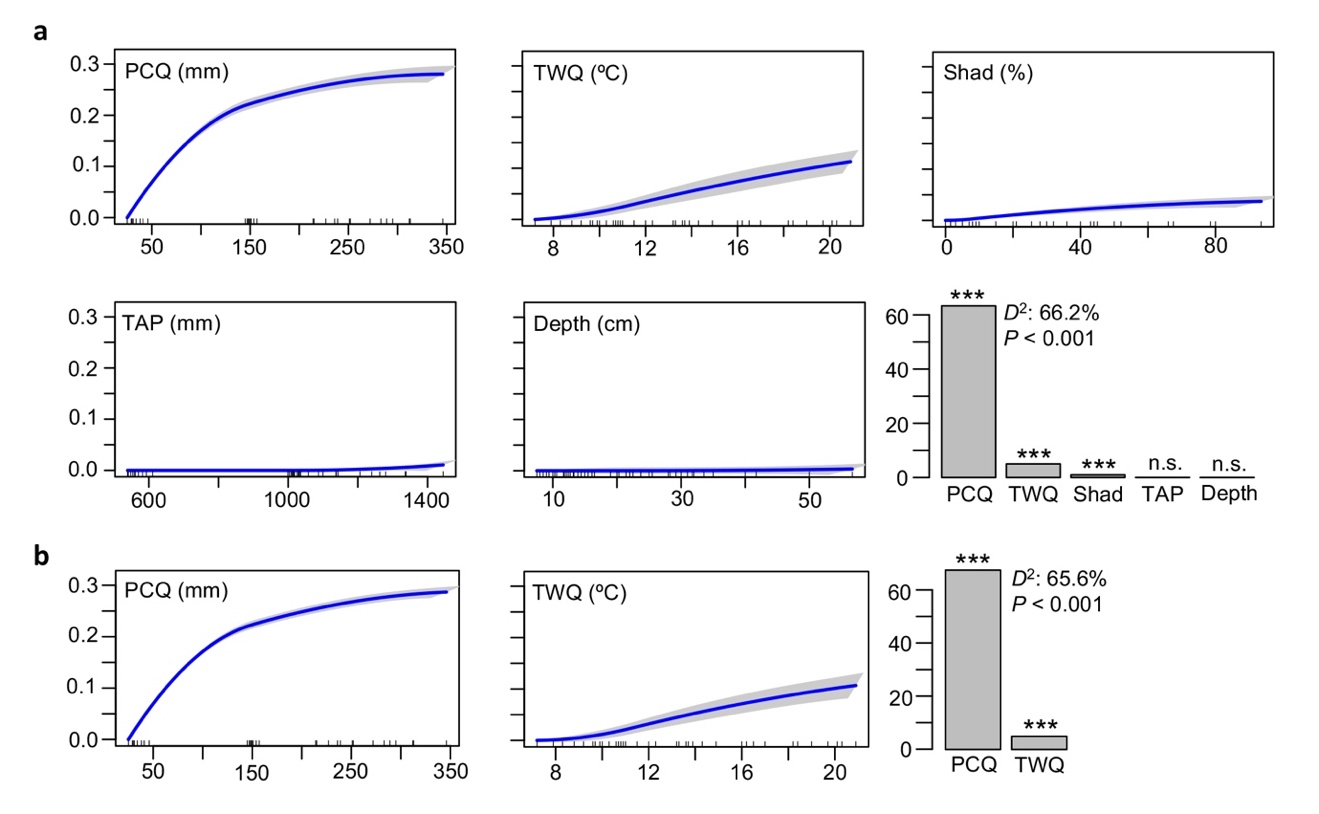


Figure S6. I-splines of predictors (blue) with their confidence intervals from bootstrapping (grey) and importance of individual predictors, calculated by generalized dissimilarity models on the overall functional gene pool across the three mountains for the candidate model (a) and for the model employed in the projections (b). Explained deviance (*D*^2^) and *P* values of models are shown. Significance of individual predictors are indicated by *P** < 0.05; *P*** < 0.01; *P**** < 0.001. PCQ, precipitation of the coldest quarter; TWQ, mean temperature of the warmest quarter; TAP, total annual precipitation; Shad, shading.


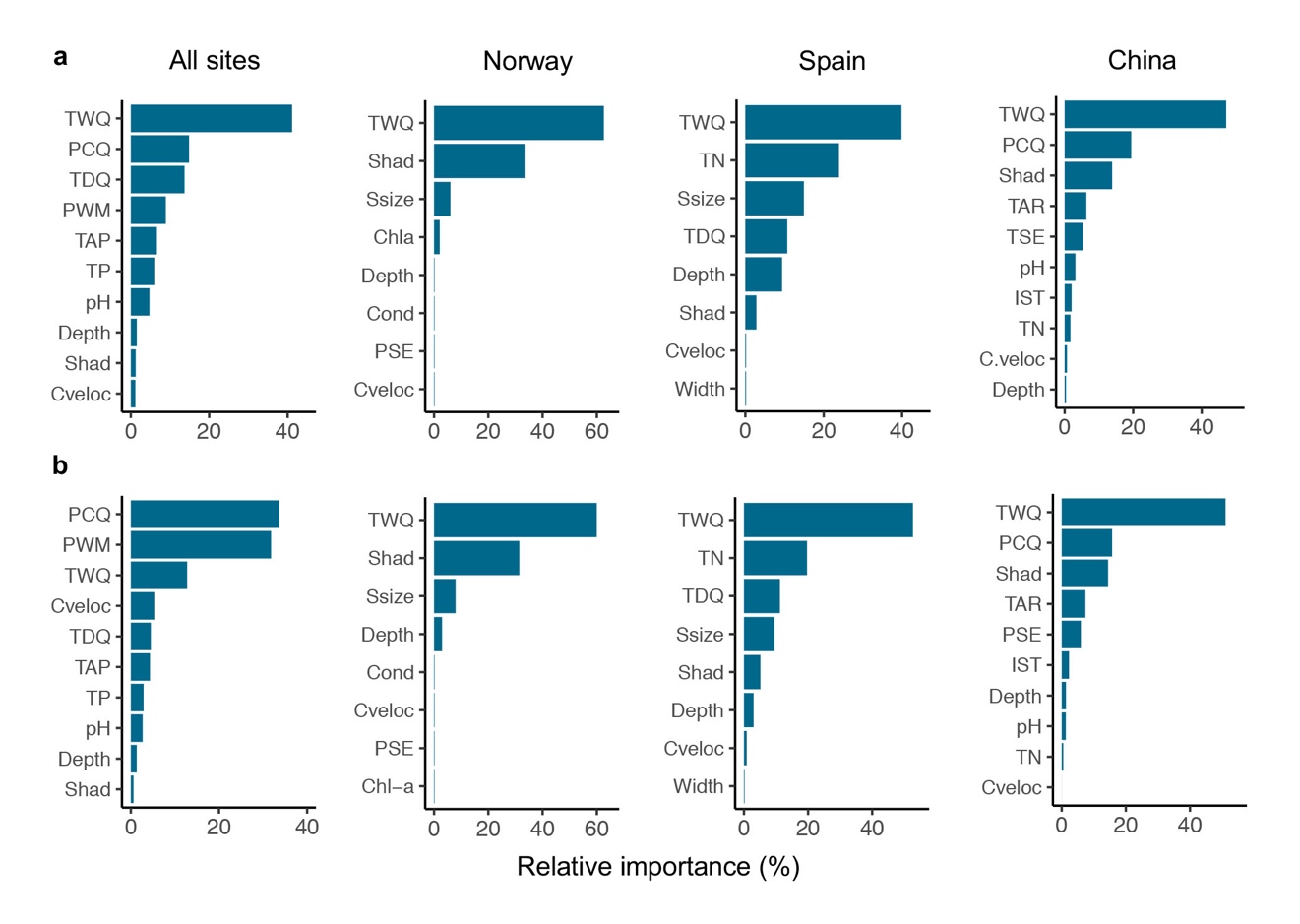


Figure S7. Relative importance of predictors considered for the full models in explaining overall alpha diversity (a) and assemblage composition (b) on the whole study extent and individual mountains, calculated by random forest analyses. Alpha diversity was calculated as the Shannon-Wiener index and assemblage composition was approached from the first axis of principal coordinate analysis (Bray-Curtis dissimilarity). TWQ, mean temperature of the warmest quarter; PCQ, precipitation of the coldest quarter; TDQ, mean temperature of the driest quarter; PWM, precipitation of the wettest month; TAP, total annual precipitation; PSE, precipitation seasonality; TAR, temperature annual range; TSE, temperature seasonality; IST, isothermality; TP, total phosphorus; Shad, shading; Cveloc, current velocity; Ssize, stone size; Cond, conductivity; Chl-a, chlorophyll-*a*; TN, total nitrogen.


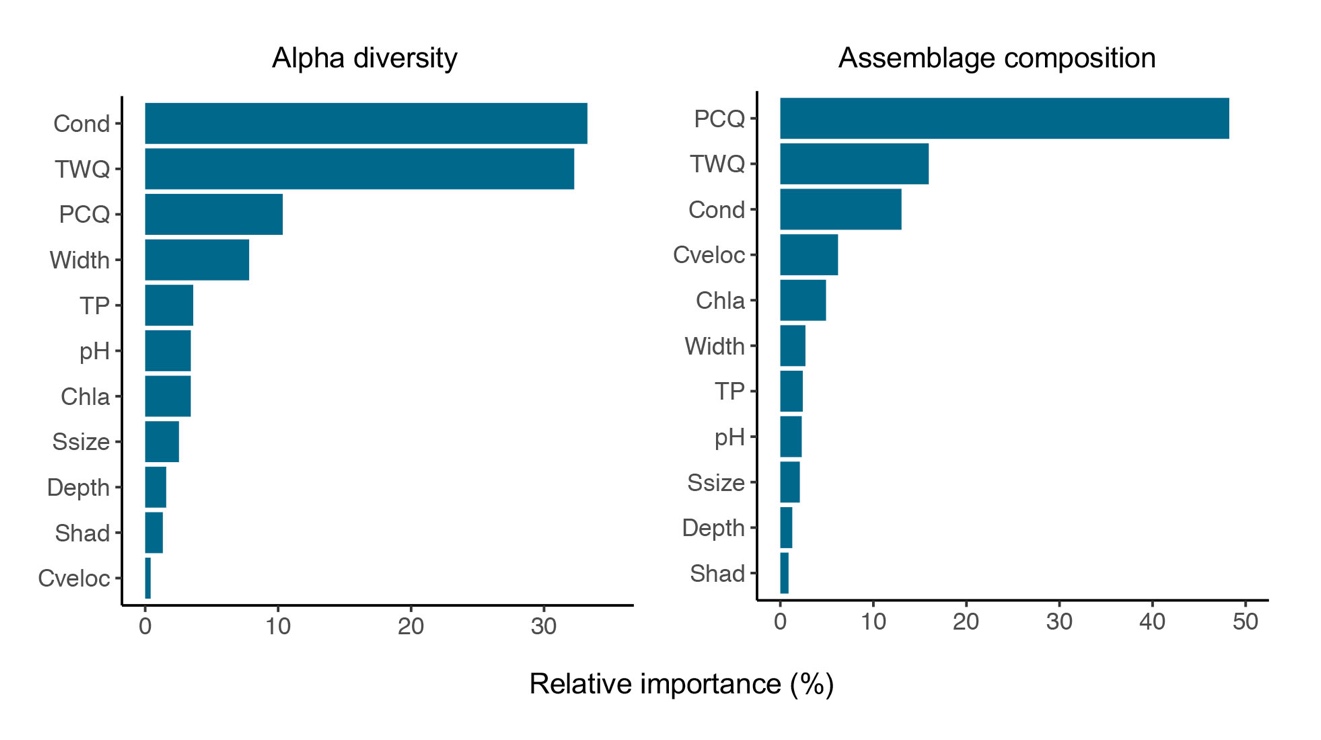


Figure S8. Relative importance of predictors in explaining overall alpha diversity and assemblage composition for the whole study extent, assessed by random forest analyses. We considered the best bioclimatic predictors of alpha diversity (temperature of the warmest quarter) and assemblage composition (precipitation of the coldest quarter) as well as all local non-climatic predictors irrespective of their correlation with the rest of predictors. Alpha diversity was calculated as the Shannon-Wiener index and assemblage composition was approached from the first axis of principal coordinate analysis (Bray-Curtis dissimilarity). TWQ, mean temperature of the warmest quarter; PCQ, precipitation of the coldest quarter; Cond, conductivity; TP, total phosphorus; Chl-a, chlorophyll-*a*; Ssize, stone size; Shad, shading; Cveloc, current velocity.


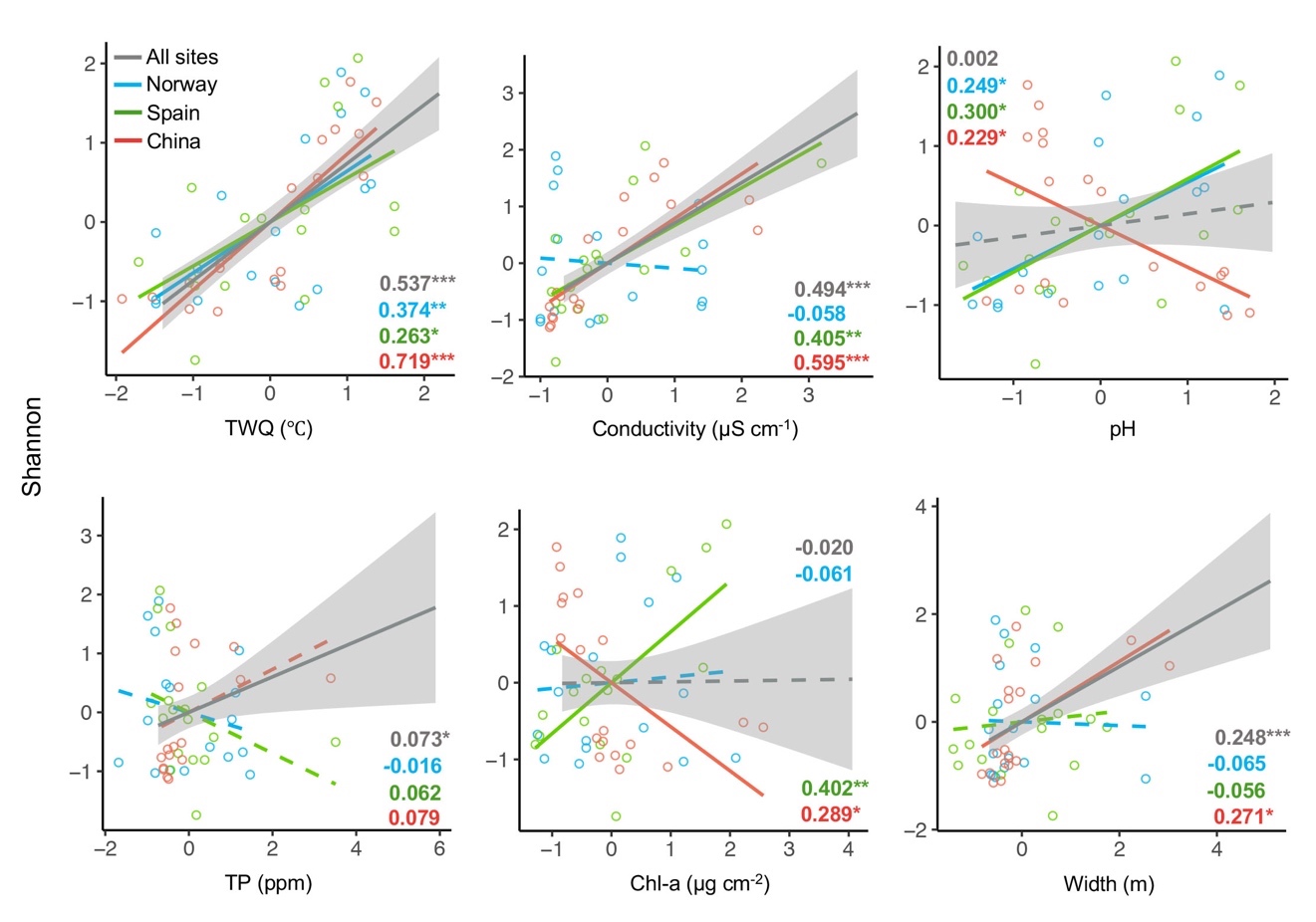


**Figure S9.** Alpha diversity response to main climatic and local non-climatic predictors, calculated by linear models on z-transformed values for the whole study extent and sites from each mountain. Alpha diversity was calculated as the Shannon-Wiener index. Inset values correspond to the adjusted *R*^2^, with colours denoting their respective data sets. The significance level is indicated by **P* < 0.05, ***P* < 0.01, ****P* < 0.001 and non-significant trends are denoted by dashed lines. The 95% confidence interval is also shown (shaded area) for those models based on the whole study extent. TWQ, temperature of the warmest quarter; TP, total phosphorus; Chl-a, chorophyll-*a*.


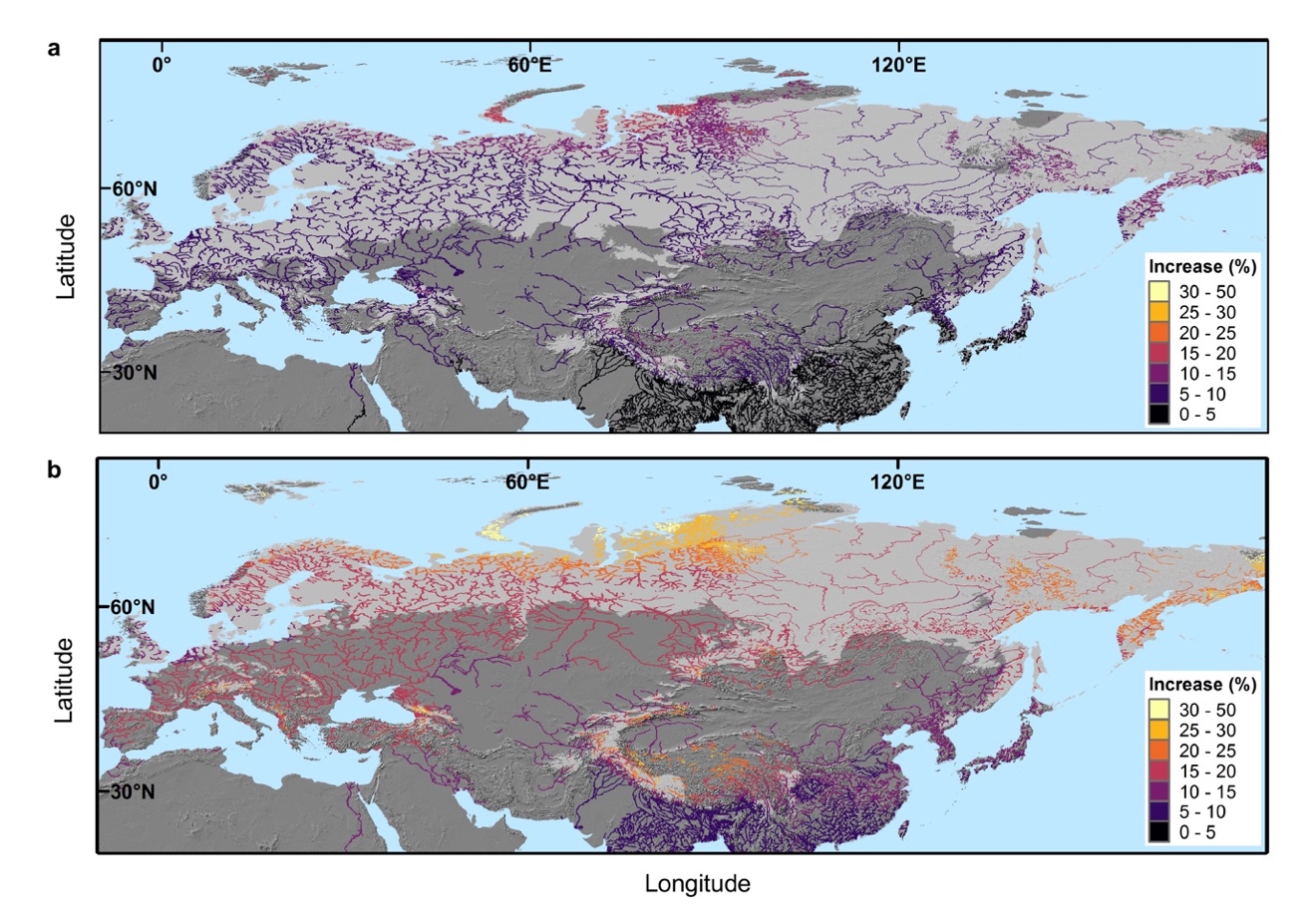


Figure S10. The projected changes in alpha diversity (Shannon-Wiener index) across the Eurasian river network, calculated from linear models with mean temperature of the warmest quarter (TWQ) and precipitation of the coldest quarter (PCQ) as predictors, under the representative concentration pathways RCP 2.6 (a) and RCP 8.5 (b). Light and dark grey areas depict the climatic envelope covered by and extrapolated from the *in* *situ* data, respectively.


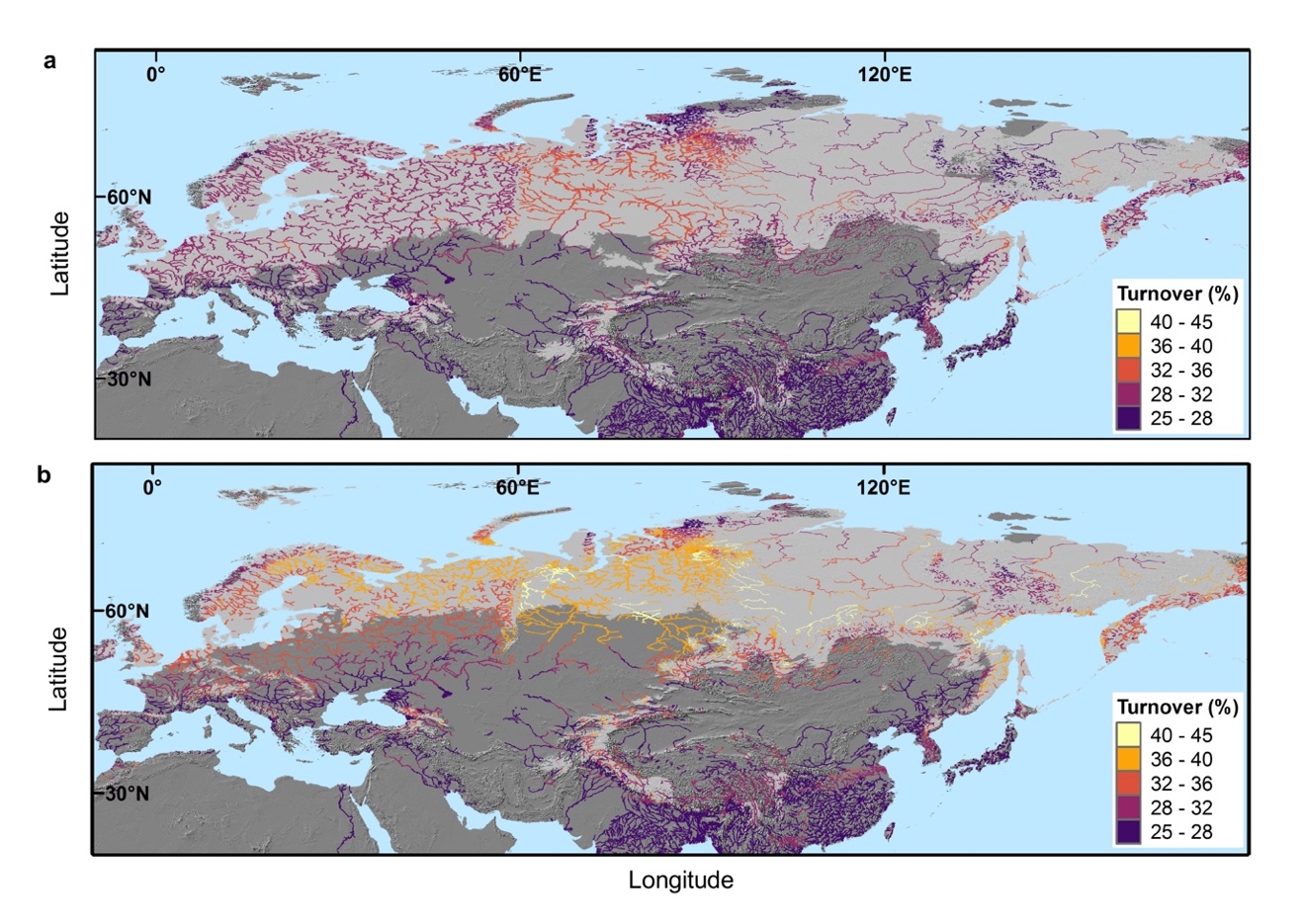


Figure S11. The projected turnover rates (Bray-Curtis dissimilarity) across the Eurasian river network, calculated from generalized dissimilarity models with mean temperature of the warmest quarter (TWQ) and precipitation of the coldest quarter (PCQ) as predictors, under the representative concentration pathways RCP 2.6 (a) and RCP 8.5 (b). Light and dark grey areas depict the climatic envelope covered by and extrapolated from the *in* *situ* data, respectively.


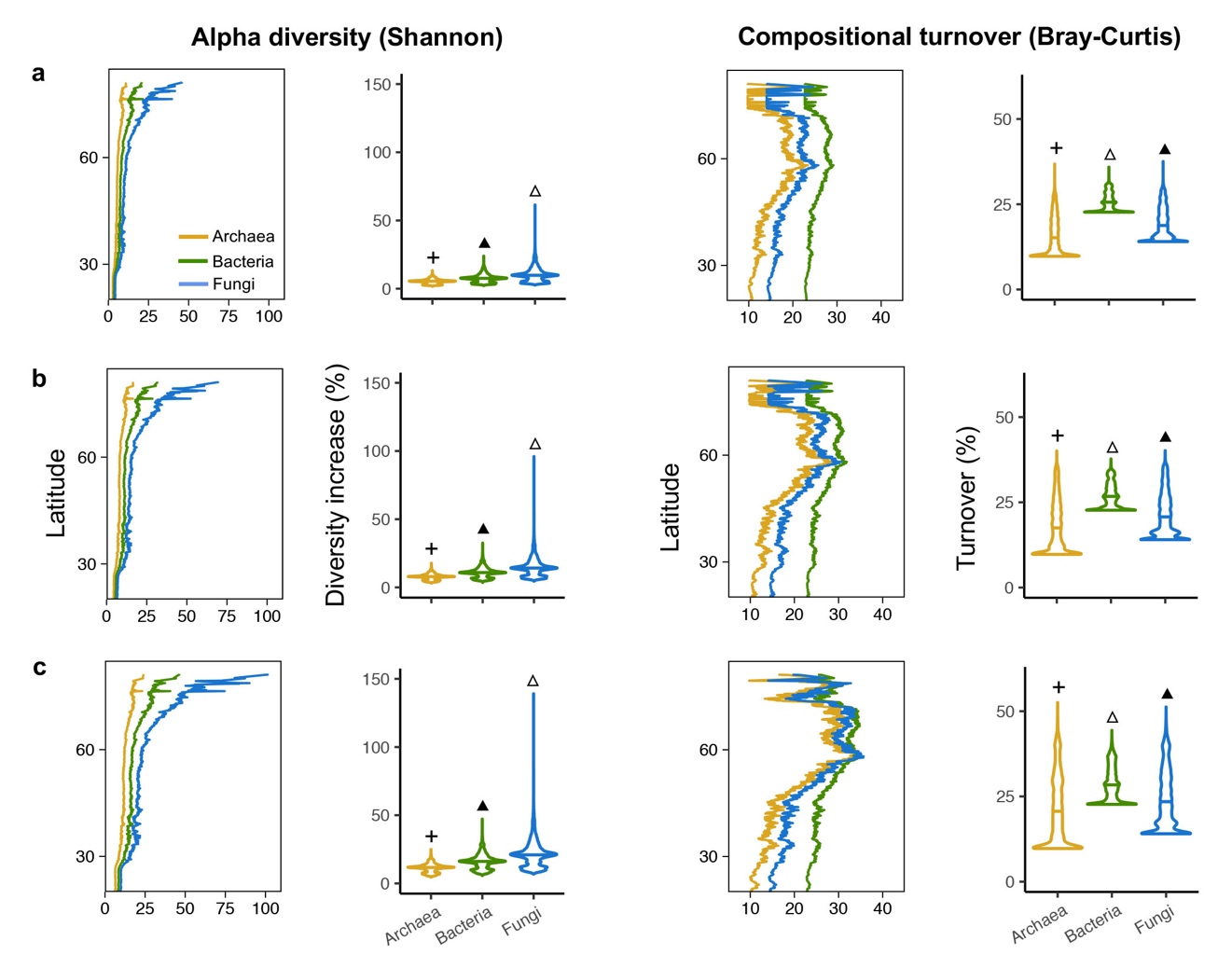


Figure S12. Projected changes across the Eurasian river network in functional gene alpha diversity and assemblage composition under the emission scenarios RCP 2.6 (a), RCP 4.5 (b) and RCP 8.5 (c) for archaea, bacteria and fungi. The relative increase in alpha diversity (Shannon-Wiener index; left panels, line plots) averaged by latitude for the three emission scenarios were calculated by linear models using temperature of the warmest quarter (TWQ) and precipitation of the coldest quarter (PCQ) as predictors (archaea, *R*^2^ = 0.478, *P* < 0.001; bacteria, *R*^2^ = 0.544, *P* < 0.001; fungi, *R*^2^ = 0.531, *P* < 0.001). The turnover rates (Bray-Curtis dissimilarity; right panels, line plots) averaged by latitude for the three emission scenarios were calculated using generalized dissimilarity models using TWQ and PCQ as predictors (archaea, *D*^2^ = 75.1%, *P* < 0.001; bacteria, *D*^2^ = 65.6%, *P* < 0.001; fungi, *D*^2^ = 50.1%, *P* < 0.001). The violin boxplots show the median and the first and the third quartiles for the relative increase in alpha diversity (left panels) and the turnover rate (right panels). Pairwise differences across kingdoms regarding the relative change in alpha diversity and turnover rates were examined by a Bonferroni-corrected pairwise *t*-test (*P* < 0.05) post hoc analyses and are indicated with the symbols +, ▲ and △.


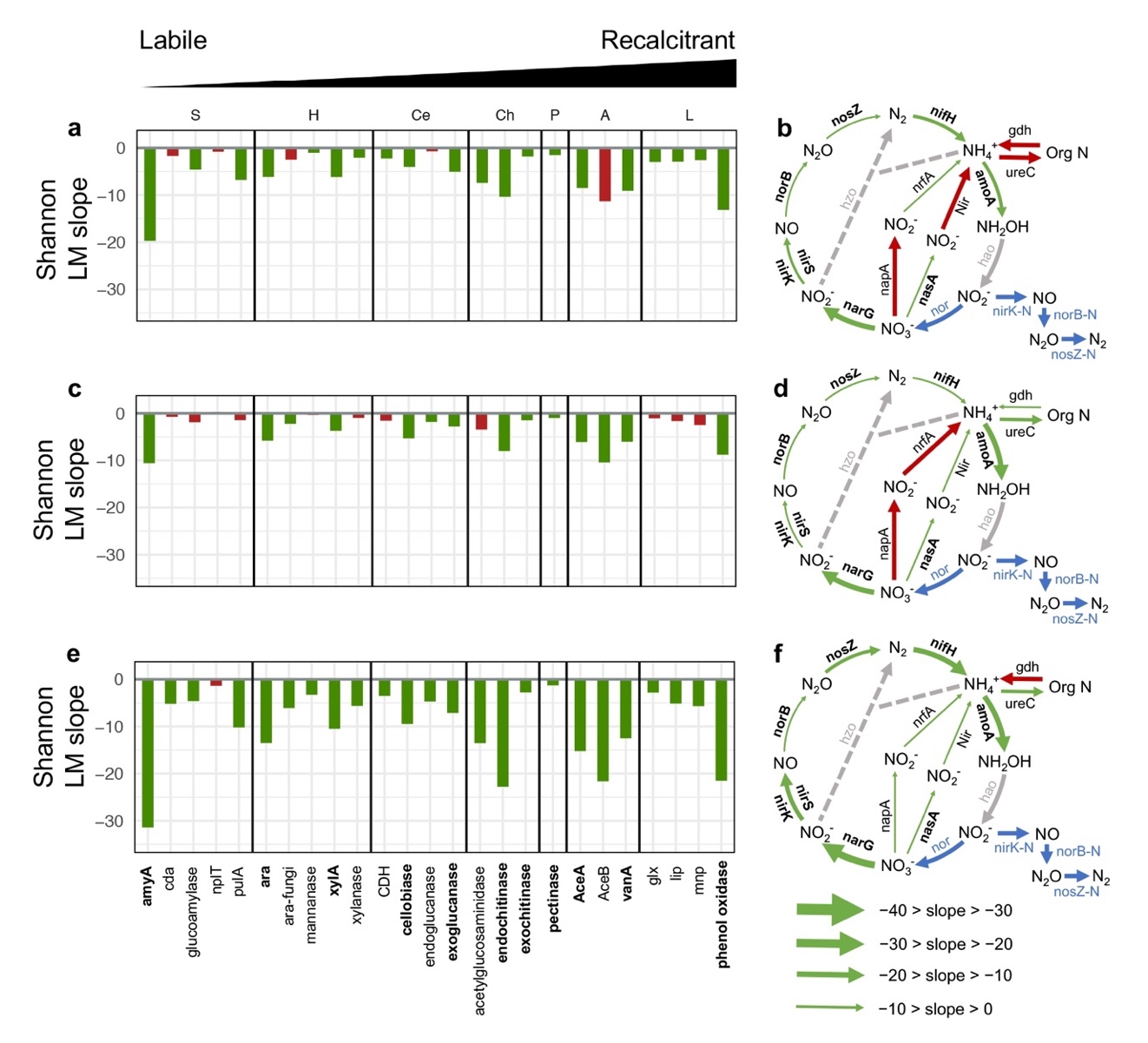


Figure S13. The alpha diversity (Shannon-Wiener index) response to elevation, calulated from linear model slopes, for gene families associated with the cycling of carbon (left panels) and nitrogen (right panels) in Norway (a, b), Spain (c, d) and China (e, f). The significant (*P* < 0.05) and non-significant (*P* > 0.05) relationships are shown in green and red, respectively. Gene families in bold (along the x-axis for carbon and aside the arrow for nitrogen) indicate patterns which are consistently significant across the three mountains. For nitrogen, arrow thickness is proportional to the slope values, functional gene families in grey denote those excluded from the analyses as they belong to the lowest functional gene richness quartile and gene families in blue denotes they were not detected or not applicable in the employed version of GeoChip. Carbon degradation subcategories: S, starch; H, hemicellulose; Ce, cellulose; Ch, chitin; P, pectin; A, aromatics; L, lignin.


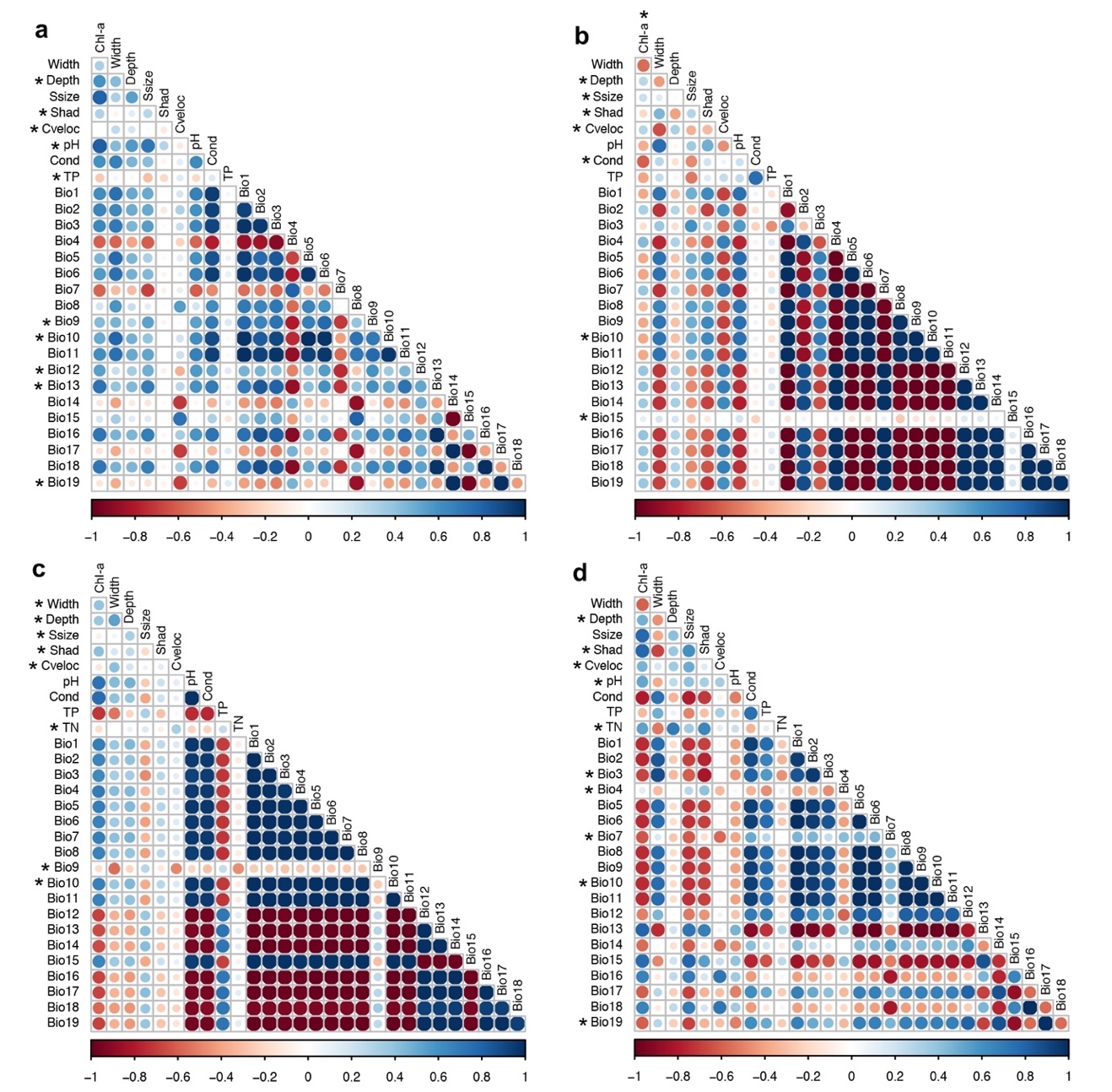


Figure S14. Spearman correlations on all climatic and local non-climatic predictors for the whole study extent (a) and the mountains in Norway (b), Spain (c) and China (d). Asterisks indicate the pre-selected climatic and non-climatic predictors included in the multi-model approach for each dataset (see Materials and methods for details). Colours depict the correlation values. Chl-a, chlorophyll-*a*; Ssize, stone size; Shad, shading; Cveloc, current velocity; Cond, conductivity; TP, total phosphorus; Bio1, anual mean temperature; Bio2, mean diurnal range; Bio3, isothermality; Bio4, temperature seasonality; Bio5, max temperature of warmest month; Bio6, min temperature of coldest month ; Bio7, temperature annual range; Bio8, mean temperature of wettest quarter; Bio9, mean temperature of driest quarter; Bio10, mean temperature of warmest quarter; Bio11, mean temperature of coldest quarter; Bio12, annual precipitation; Bio13, precipitation of wettest month; Bio14, precipitation of wettest month; Bio15, precipitation seasonality; Bio16, precipitation of wettest quarter; Bio17, precipitation of driest quarter; Bio18, precipitation of warmest quarter; Bio19, precipitation of coldest quarter.
